# Key Advancements and Emerging Trends of Perovskite Solar Cells in 2024–2025

**DOI:** 10.1007/s40820-025-02022-6

**Published:** 2026-01-15

**Authors:** Xiangqian Shen, Xuesong Lin, Hongzhen Su, Ziyang Zhang, Tianhao Wu, Jing Zhang, Yong Peng, Yiqiang Zhang, Shufang Zhang, Zhongmin Zhou, Xiangyue Meng, Peng Gao, Wei Chen, Yongzhen Wu, Chuanjiang Qin, Qifeng Han, Yanbo Wang, Liyuan Han

**Affiliations:** 1 Xinjiang Key Laboratory of Solid State Physics and Devices, School of Physical Science and Technology, Xinjiang University, Urumqi, 830046 People’s Republic of China; 2 Present Address: State Key Laboratory of Metal Matrix Composites, Shanghai Jiao Tong University, Shanghai, 200240 People’s Republic of China; 3 Materials Genome Institute (MGI), Shanghai University, Shanghai, 200444 People’s Republic of China; 4 School of Physical Science and Technology, Ningbo University, Ningbo, 315211 People’s Republic of China; 5 State Key Laboratory of Advanced Technology for Materials Synthesis and Processing, Wuhan University of Technology, Wuhan, 430070 People’s Republic of China; 6 College of Chemistry, Henan Institute of Advanced Technology, Zhengzhou University, Zhengzhou, 450001 People’s Republic of China; 7 School of Physics and Optoelectronic Engineering, Ludong University, Yantai, 264025 People’s Republic of China; 8 College of Chemistry and Molecular Engineering, Qingdao University of Science and Technology, Qingdao, 266042 People’s Republic of China; 9 School of Optoelectronics, Center of Materials Science and Optoelectronics Engineering, University of Chinese Academy of Sciences, Beijing, 100049 People’s Republic of China; 10 State Key Laboratory of Structural Chemistry, Fujian Institute of Research on the Structure of Matter, Chinese Academy of Sciences, Fuzhou, 350002 People’s Republic of China; 11 Wuhan National Laboratory for Optoelectronics (WNLO), Huazhong University of Science and Technology, Wuhan, 430074 People’s Republic of China; 12 School of Chemistry and Molecular Engineering, East China University of Science and Technology, Shanghai, 200237 People’s Republic of China; 13 Changchun Institute of Applied Chemistry, Chinese Academy of Sciences, Changchun, 130022 People’s Republic of China

**Keywords:** Perovskite solar cells, Tandem architectures, Interface engineering, Operational stability, Scalable fabrication

## Abstract

The past two years have witnessed remarkable progress in perovskite solar cells (PSCs), marked by breakthroughs in power conversion efficiency and strides in addressing long-term operational stability. At present, the certified power conversion efficiencies of single-junction PSCs and silicon/perovskite tandem cells have surpassed 27% and 34%, respectively. Regarding stability, researchers begun to focus their attention on the challenges faced by PSCs when operated in outdoor environments. Furthermore, breakthroughs in the utilization of green solvents, fabrication in ambient air conditions, aqueous-phase synthesis of perovskite raw materials at kilogram scale, vacuum flash evaporation, and machine learning-assisted design are accelerating the commercialization of PSCs. The review summarizes the key advancements of PSCs during 2024–2025. It identifies a critical performance discrepancy between small-area devices and perovskite solar modules and delves into strategies aimed at bridging this gap. Finally, perspectives on the future directions of PSCs are presented, with a particular emphasis on improving photocurrent and environmental sustainability.

## Highlights

- The key advancements in perovskite solar cells during the years 2024–2025 are summarized, along with an in-depth exploration of the underlying enhancement mechanisms.
- The performance gap between small-area devices and perovskite solar modules is highlighted.
- The future directions aimed at accelerating the commercialization and enhancing the sustainability of perovskite solar cells are provided.

## Graphical abstract

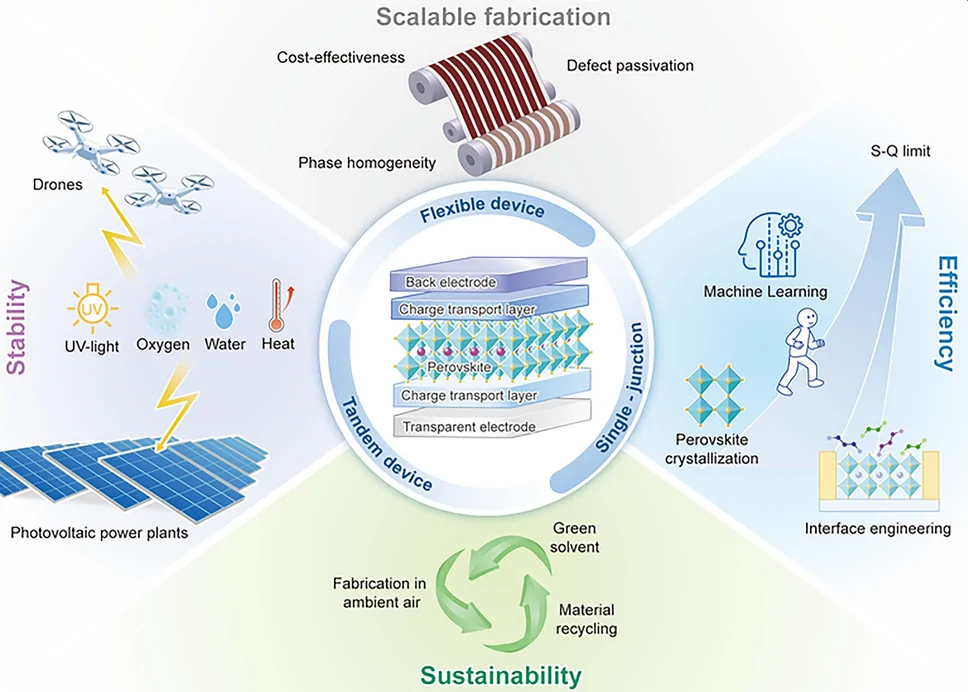

## Introduction

In the quest for renewable energy sources to mitigate the adverse impacts of fossil fuel consumption and global warming, perovskite solar cells (PSCs) have emerged as a promising candidate due to their exceptional optoelectronic properties, low manufacturing costs, high power conversion efficiencies (PCEs), and potential for lightweight and flexible applications [1–6]. Currently, PSCs have rivaled crystalline silicon solar cells in terms of PCE [7, 8]. However, the journey from laboratory breakthroughs to commercial viability hinges on resolving critical challenges: long-term operational stability under outdoor conditions [9–12], scalable manufacturing of high-performance large-area modules [6, 13–15], and ensuring environmental sustainability [16–19]. Addressing these challenges with practical solutions is crucial as PSCs mature beyond proof of concept. Notably, the past two years have marked a critical inflection point for PSC research, with their PCEs being continually updated to new benchmarks, and efforts increasingly oriented toward bridging the gap between laboratory innovation and market readiness.

At present, the certified PCEs of single-junction PSCs have exceeded 27% [8]. Self-assembled monolayers (SAMs), which are utilized as hole transport layers (HTLs) in inverted PSCs, have played a pivotal role in boosting the PCE of these devices [20–22]. The coverage of SAMs on transparent conducting oxide (TCO) substrates, along with the bonding and stability of SAMs at buried interfaces, represents a key area of research focus within this field [23–27]. Benefiting from the optimization of device interfaces via SAMs, the certified PCE of inverted PSCs surpassed that of their regular counterparts for the first time in 2024 [28]. In the same year, the steady-state PCE of perovskite/silicon tandem solar cells reached 33.89%. This marks the first reported certification of a dual-junction tandem solar cell surpassing the single-junction Shockley–Queisser limit of 33.7% [29]. Recently, the certified PCE of such tandem devices has broken through the 34% mark. Additionally, researchers have dedicated considerable efforts to narrowing the notorious "PCE gap" that exists between small-area PSCs and perovskite solar modules (PSM) [30, 31]. Studies demonstrated that meticulous control of homogeneity, pinhole density, and charge collection losses during scaling can yield a PCE of 22.46% for the PSM with an aperture area of 715.1 cm^2^ [32].

The Achilles' heel of PSCs remains their susceptibility to degradation under operating conditions. In the past two years, significant strides have been made in enhancing the stability of PSCs through material innovation and device architecture optimization [33, 34]. For instance, the incorporation of additives and passivating agents within the perovskite lattice has been shown to mitigate ion migration and defect formation, thereby improving resistance to humidity and thermal stress [35, 36]. Additionally, the formation of two-dimensional (2D)/three-dimensional (3D) heterojunctions through the combination of 2D or quasi-2D perovskites with 3D perovskites has attracted considerable attention [37–39]. High-performance devices can withstand aging tests such as maximum power point tracking (MPPT) under 85 °C and 85% relative humidity (RH) environment, as well as standard test protocols including International Electrotechnical Commission (IEC) and International Summit on Organic Photovoltaic Stability (ISOS) [40, 41]. Furthermore, emphasis is now placed on investigating the operational stability of PSCs under day–night cycling conditions [42]. It is commendable that PSMs demonstrated an operational PCE exceeding 16% for 29 weeks under outdoor conditions [43]. Encouragingly, after one year of operation, a 0.5-megawatt PSM system achieved a higher energy yield per kilowatt of installed capacity in comparison with silicon modules [44].

The scalability of PSC production is another pivotal factor for their commercialization. Recent advancements demonstrated the feasibility of fabricating large-area PSCs with minimal PCE loss. Techniques such as vacuum flash evaporation, blade coating, slot-die coating, and roll-to-roll processing have emerged as promising approaches for scalable manufacturing [6, 14, 30, 45, 46]. These methods not only enable high-throughput production but also facilitate cost reduction by minimizing material waste and simplifying the manufacturing process. In parallel, efforts to optimize the uniformity and quality of perovskite films over large areas have been intensified, including the use of advanced deposition techniques and post-treatment strategies to ensure consistent device performance across different scales [47–49]. In pursuit of cost-effectiveness and sustainability, the development of green solvents and techniques for fabricating PSCs in ambient air garnered considerable attention over the two past years [13, 50]. Of particular note, researchers successfully synthesized formamidinium lead iodide (FAPbI_3_) microcrystals with an average purity of 99.994% through aqueous-phase synthesis [51]. This breakthrough has enabled the scaled production of these crystals on a kilogram scale.

Herein, this review summarizes the key advancements in PSCs during 2024–2025. It begins by focusing on PCE breakthroughs in single-junction rigid, flexible, and tandem solar cells. Subsequently, the underlying reasons for performance enhancements are explored from the perspectives of interface engineering, charge transport layer (CTL) design, and perovskite crystallization. Furthermore, we highlight PSC stability and large-area fabrication techniques. Finally, the conclusions and future directions aimed at paving the way for the commercialization of PSCs are outlined.

## Single-Junction Perovskite Solar Cells

At present, the certified PCEs of small-area and centimeter-square-scale single-junction PSCs have surpassed 27% and 25%, respectively. More encouragingly, the PCE of PSMs reached ~ 23%, further narrowing the PCE gap with small-area devices [52]. These accomplishments are attributed to the diligent efforts of researchers in areas such as interface engineering, CTL design, and perovskite crystallization. In addition, artificial intelligence (AI), particularly machine learning (ML), is increasingly prominent in enhancing the performance of PSCs. Furthermore, the certified PCE of flexible perovskite solar cells (FPSCs) reached ~ 25%, accompanied by a notably power per weight 44.1 W g^−1^, which renders them promising candidates for applications in fields such as drones [53, 54]. Single-junction PSCs with outstanding-certified PCEs are summarized in Table 1.

**Table 1:** Summary of certified photovoltaic parameters of single-junction PSCs

Device structure	Area(cm^2^)	*V*_OC_ (V)	*J*_SC_ (mA cm^− 2^)	FF (%)	PCE (%)	Certified PCE (%) /Institution	References
ITO/NiO_X_/D4PA/Cs_0.05_FA_0.85_MA_0.1_PbI_3_/C_60_/BCP/Ag	0.04	1.197	26.2	85.17	26.83	26.72/NPVM	[26]
ITO/SnO_2_/FA_0.94_Cs_0.06_PbI_3_/Spiro-OMeTAD/Au	0.049	1.21	25.69	85	26.41	25.94/NPVM	[55]
FTO/2PACz/Me-4PACz/Cs_0.05_MA_0.1_FA_0.85_PbI_3_/C_60_/SnO_x_/Ag	0.05	1.18	26.4	86.2	26.9	26.15/Newport	[28]
FTO/2PACz/4PACz/Cs_0.05_MA_0.05_FA_0.90_PbI_3_/C_60_/SnO_2_/Cu	0.05	1.18	26.5	85.5	26.7	26.3/NPVM	[56]
FTO/SnO_2_/FAPbI_3_/Spiro-OMeTAD/Au	0.0526	1.179	26.3	85.8	26.61	26.54/NPVM	[57]
ITO/MeO-2PACz/ Rb_0.05_Cs_0.05_MA_0.05_FA_0.85_Pb(I_0.95_Br_0.05_)_3_/PC_61_BM/BCP/Ag	0.053	1.19	26.2	85.3	26.6	26.44/IEE, CAS	[27]
ITO/4PADCB/Al_2_O_3_/FA_0.95_Cs_0.05_PbI_3_/PI/PCBM/BCP/Ag	0.053	1.192	26.558	84.93	27.02	26.88/ IEE, CAS	[58]
FTO/NiO_X_/Me-4PACz/FA_0.95_Cs_0.05_PbI_3_/PC_61_BM/BCP/Ag	0.053	1.18	26.27	86.06	–	26.73/TIMST	[59]
ITO/NiO_X_/Me-4PACz/ Cs_0.05_MA_0.05_FA_0.9_PbI_3_/C_60_/BCP/Ag	0.055	1.206	26.34	85.59	27.18	26.79/TIMST	[60]
ITO/NiO_X_/Me-4PACz/FA_0.95_Cs_0.05_PbI_3_/PC_61_BM/BCP/Ag	0.057	1.201	26.3	84.5	26.69	26.54/NPVM	[61]
FTO/SnO₂/FAPbI₃/Spiro-OMeTAD/Au	0.0583	1.187	26.2	84.55	–	26.32/SIMIT	[62]
FTO/c-TiO_x_/m-TiO_x_/FAPbI_3_/Spiro-OMeTAD/Au	0.07	1.175	26.275	85.9	26.52	26.31/NIM	[63]
ITO/2-PACz/FA_0.85_MA_0.1_Cs_0.05_PbI_3_/C_60_/BCP/Cu	0.08	1.19	25.02	86.0	25.6	25.3/SIMIT	[51]
ITO/Py3/(FA_0.98_MA_0.02_)_0.95_Cs_0.05_Pb(I_0.98_Br_0.02_)_3_/C_60_/BCP/Ag	0.09	1.18	26	85.1	26.1	25.7/SIMIT	[64]
ITO/MeO-2PACz/Me-4PACz/CsFAMA/C_60_/BCP/Ag	0.1	1.17	25.8	85.2	25.7	25.5/SIMIT	[65]
ITO/HTM/Cs_0.05_MA_0.1_FA_0.85_PbI_3_/PCBM/BCP/Ag	0.1	1.195	26.01	84.35	26.2	25.9/SIMIT	[66]
FTO/SnO_2_/Cs_0.05_MA_0.05_FA_0.9_PbI_3_/Spiro-OMeTAD/Au	1	1.15	26.2	83.2	25.1	24.6/Newport	[37]
FTO/Ph-4PACz/Cs_0.1_FA_0.9_PbI_3_/C_60_/BCP/Ag	1	1.16	25.6	82.0	25.20	24.35/NPVM	[67]
ITO/NiO_X_/MDA/Cs_0.05_FA_0.85_MA_0.1_PbI_3_/C_60_/BCP/Ag	1.02	1.19	25.27	82.81	25.31	24.9/SIMIT	[68]
FTO/2PACz/Me-4PACz/Cs_0.05_MA_0.1_FA_0.85_PbI_3_/C_60_/SnO_x_/Ag	1.04	1.167	26.45	80.1	–	24.74/Newport	[28]
FTO/TiO_2_/SnO₂/Cs_0.05_MA_0.05_FA_0.90_Pb(I_1−x_Cl_x_)_3_/Spiro-OMeTAD/MoO₃/ITO/Au	27.22	9.402	83.96	80.33	23.2	23.3/NPVM	[69]
FTO/TiO₂/SnO₂/FAPbI₃/Spiro-OMeTAD/MoOₓ/Cu	715.1	49.51	0.557	82.68	22.8	22.46/NPVM	[32]
PEN/ITO/SnO_2_/FAPbI_3_/Spiro-OMeTAD/Ag	0.08	1.20	24.91	81.79	24.51	24.04/SIMIT	[70]
PET/ITO/SnO_2_/Perovskite/Spiro-OMeTAD/Au	0.1	1.18	25.29	83.5	25.09	24.9/NIM	[53]
PET/ITO/PTAA/MA_0.7_FA_0.3_PbI_3_/C_60_/BCP/Cu	9	5.85	4.26	75.48	20.1	18.8/NREL	[71]
PEN/ITO/SnO_2_/(FAPbI_3_)_0.95_(MAPbBr_3_)_0.05_/Spiro-OMeTAD/Au	900	54	0.488	62.36	–	16.43/CBTP	[72]

NPVM: National PV Industry Measurement and Testing Center, China; Newport: USA; IEE, CAS: Institute of Electrical Engineering, Chinese Academy of Sciences, China; TIMST: Tianjin Institute of Metrological Supervision and Testing, China; NIM: National Institute of Metrology, China; SIMIT: Shanghai Institute of Microsystem and Information Technology, China; NREL: National Renewable Energy Laboratory, USA; CBTP: Chungbuk Technopark, Korea

### Interface Engineering

**Fig. 1:**
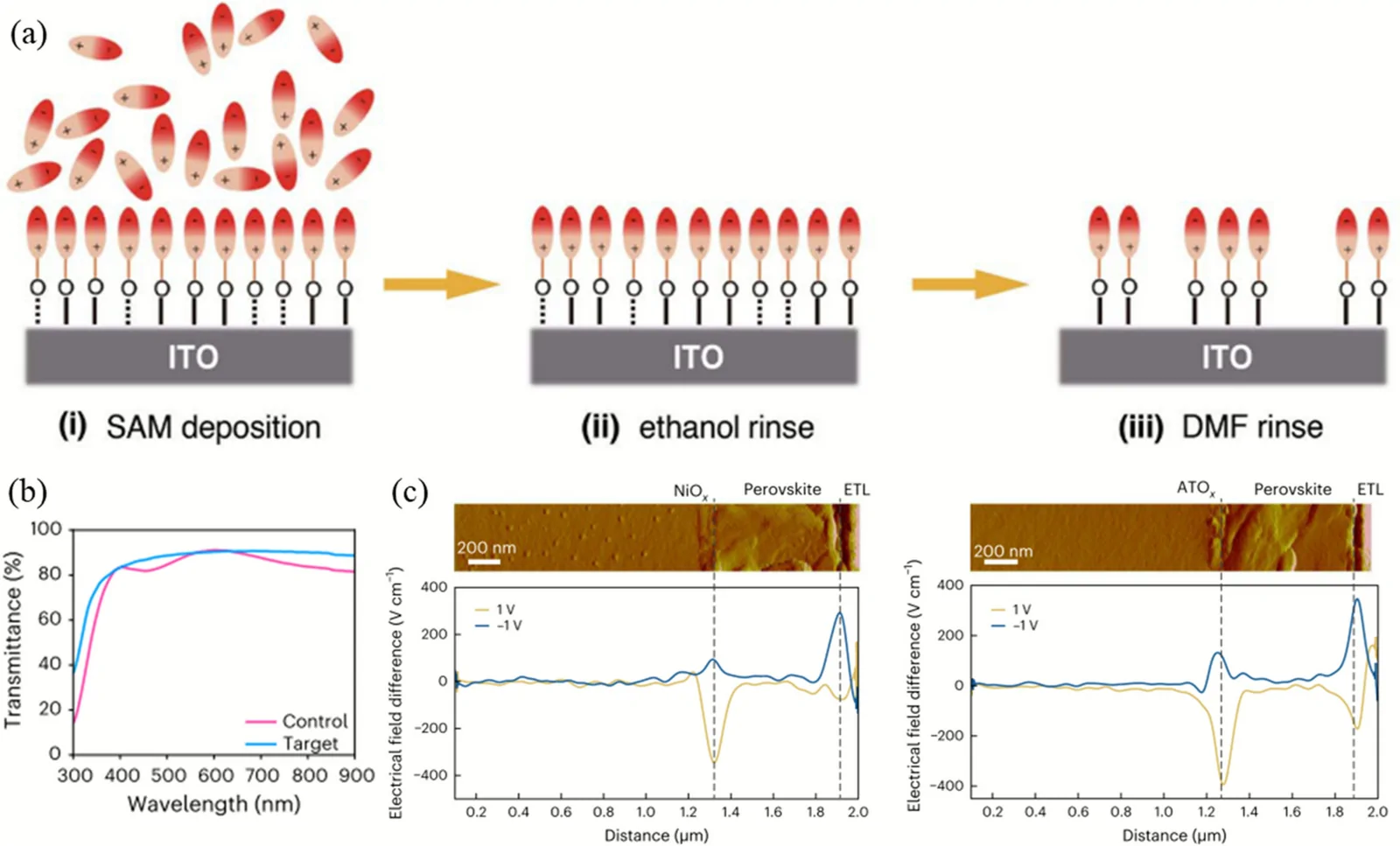
**a** Schematic illustration of the deposition of SAMs on the surface of ITO and their desorption after rinsing with ethanol and DMF. Dotted line represents a weak hydrogen bond. Reproduced with permission from Ref. [75]. Copyright 2024, AAAS. **b** Transmittance of various ITO substrates. Reproduced with permission from Ref. [27]. Copyright 2025, Springer Nature. **c** Cross-sectional kelvin probe force microscopy spectra, along with the line profiles depicting the electrical field difference for both the NiO_x_/perovskite sample and ATO_x_/perovskite sample. Reproduced with permission from Ref. [77]. Copyright 2024, Springer Nature

SAM achieves self-assembly through anchoring to hydroxyl (–OH) groups on the surface of transparent conductive metal oxide (TCO). A uniform and dense coverage of TCO by SAM is crucial for achieving high-performance PSCs [27, 73]. However, in addition to robust chemical adsorption, the bonding between OH groups and TCO also involves weaker physical adsorption [74]. SAM anchored to the latter is unstable and prone to desorption under the scouring of strong polar perovskite solvents, such as *N,N'*-dimethylformamide (DMF). This results in portions of the TCO surface remaining uncovered by the SAM layer and leads to the generation of leakage currents at these sites, which subsequently impairs the PCE and operational stability of the device (Fig. 1 a). To address this issue, Han et al. employed atomic layer deposition (ALD) to grow an additional 9–10 nm thick layer of indium tin oxide (ITO) on commercial ITO substrates [75]. Compared to the pristine substrate, the ALD ITO surface is adorned with a higher density of covalently bound OH groups, which offers an abundance of anchoring sites for the SAM. Furthermore, they synthesized a novel molecule, [3,6-dimethoxy-9*H*-carbazol-9-yl]trimethoxyphenylsilane (DC-TMPS), which features a trimethoxysilane group. Unlike conventional SAMs, DC-TMPS anchors via a tridentate interaction with OH groups. These strategies enhance the adhesion between the HTL and the substrate. As a result, the PSCs based on ALD ITO/DC-TMPS with aperture areas of 0.08 and 1.01 cm^2^ achieved PCEs of 24.8% and 23.2%, respectively. After a 1000-h damp-heat test at 85 °C and 85% RH, the device retained 98.9% of the initial PCE. To tackle the problems of desorption and self-aggregation of SAMs when devices are exposed to high temperatures or undergo thermal cycling impacts, Liu et al. designed a self-assembled bilayer structure connected by covalent bonds [73]. Such covalent connections can effectively “anchor” the small-molecule SAM layer adsorbed on the TCO. Moreover, the unique face-oriented molecular arrangement in the upper layer demonstrates favorable adhesion characteristics with perovskite materials, thus enhancing the interfacial mechanical strength between the perovskite and the HTL. After 1200 thermal cycles from −40 to 85 °C, the prepared devices showed only a 3% degradation compared to their original PCE. Zhao et al. devised a solution-based approach to achieve complete hydroxylation of the ITO in as short a time as 15 s, thereby exposing abundant uncoordinated indium ions to serve as novel bonding sites for SAMs [27]. By forming coordination bonds, the anchoring stability of SAMs is significantly enhanced. Additionally, this method can spontaneously generate nano-scale anti-reflective structures on the ITO surface, thereby improving photon transmittance (Fig. 1 b). Ultimately, PSCs based on this strategy achieved a PCE of 26.6% and maintained 96% of their initial PCE after continuous operation for 2800 h at 65 °C. Besides, introducing a nickel oxide (NiO_x_) interlayer between the TCO substrate and SAM is also a strategy to improve the coverage of the HTL on the substrate. However, the inherent reactivity of NiO_x_ unavoidably makes the occurrence of redox reactions at the NiO_x_/perovskite interface [76]. Hou et al. adopted p-type antimony-doped tin oxides (ATO_x_) to replace NiO_x_ as the interlayer [77]. Compared to NiO_x_, the chemical stability of ATO_x_ can prevent undesired chemical reactions at the buried interface. Furthermore, ATO_x_ demonstrates superior electrical conductivity and improves the depletion region at the perovskite/HTL interface, as evidenced by conductive atomic force microscopy images and cross-sectional kelvin probe force microscopy spectra (Fig. 1 c). Therefore, the PSC with an area of 1 cm^2^ and utilizing ATO_x_/[4-(3,6-dimethyl-9*H*-carbazol-9-yl)butyl]phosphonic acid (Me-4PACz) as the HTL achieved a PCE of 24.6% (certified at 24.0%).

**Fig. 2:**
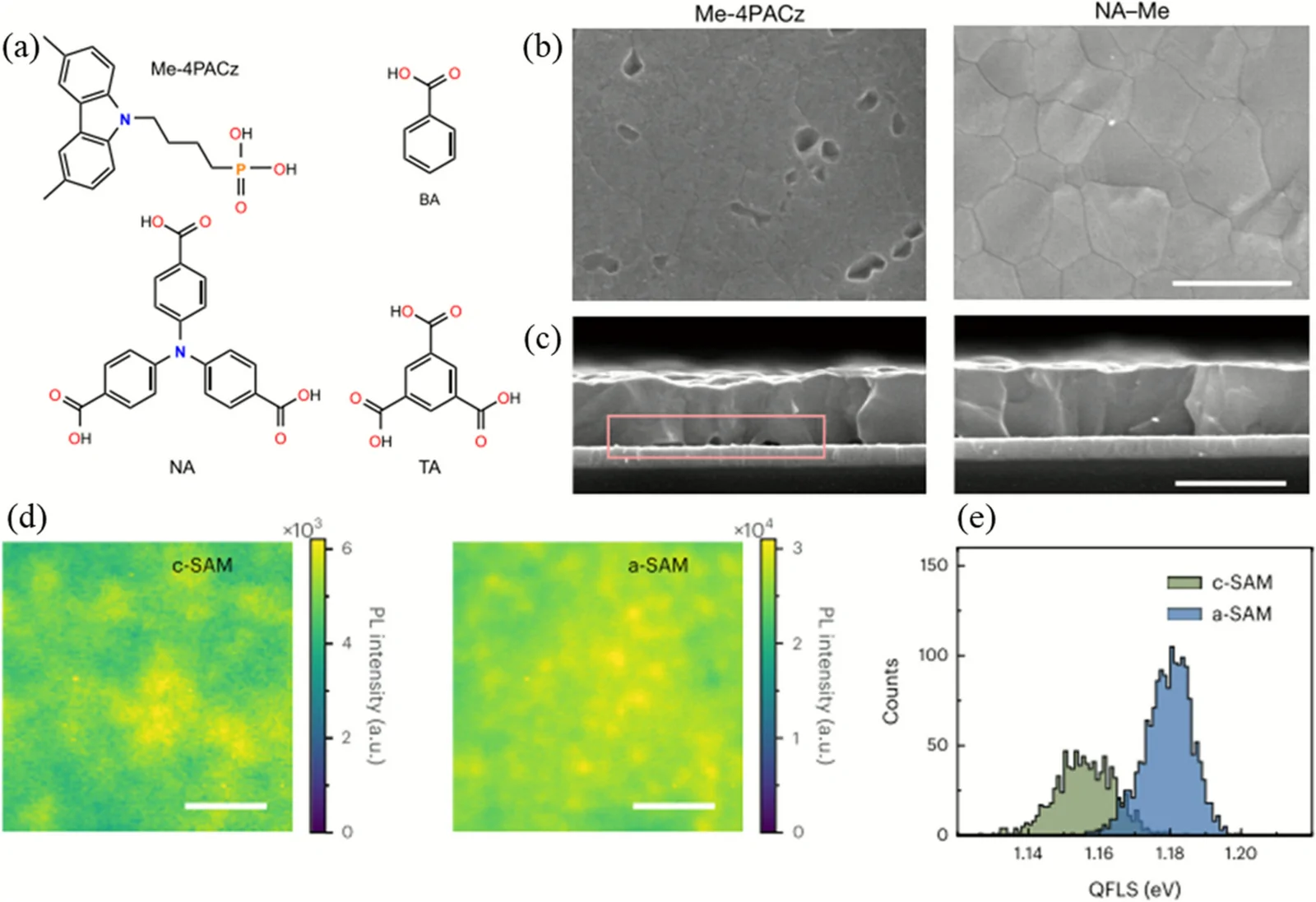
**a** Chemical structure of Me-4PACz and BA, NA, and TA. **b** Scanning electron microscope images of perovskite films deposited on various SAMs. **c** Cross-sectional scanning electron microscope images of perovskite films deposited on various SAMs. Reproduced with permission from Ref. [61]. Copyright 2024, Springer Nature.** d** Photoluminescence intensity maps for the perovskite film on c-SAM and on a-SAM. Scale bars, 10 µm. **e** Histogram of quasi-Fermi level splitting for the perovskite film on c-SAM and on a-SAM. Reproduced with permission from Ref. [67]. Copyright 2024, Springer Nature

Except for the incomplete coverage, the commonly utilized SAMs, such as Me-4PACz, also exhibit uneven distribution on TCO due to aggregation effects. Furthermore, Me-4PACz demonstrates poor surface wettability toward perovskite precursor solutions, which leads to suboptimal perovskite crystallization and the formation of numerous micro-voids at the buried interface [78]. To solve these issues, Chen et al. report a molecular hybrid strategy for the buried interface of inverted PSCs [61]. They introduced three carboxylic acid-functionalized aromatic molecules, namely 4,4',4"nitrilotribenzoic acid (NA), benzoic acid (BA), and trimesic acid (TA) into the Me-4PACz SAM precursor solution, respectively (Fig. 2 a). The resultant mixed SAMs were labeled as NA-Me, BA-Me, and TA-Me, respectively. Further research revealed that the large π-conjugated groups of NA facilitate robust π–π interactions with Me-4PACz, thereby mitigating the self-aggregation effect of Me-4PACz during deposition and inducing a more uniform distribution of Me-4PACz molecules at the microscopic scale. Moreover, the multiple carboxylic acid within NA enhances the wettability of the perovskite solution on Me-4PACz, subsequently improving the crystallinity of the perovskite (Fig. 2 b). Consequently, this leads to the elimination of nanopores at the buried interface and the release of compressive stresses within the perovskite film (Fig. 2 c). This strategy achieved optimal PCEs of 26.69% with a *V*_OC_ of 1.201 V (certified steady-state PCE of 26.54%). Furthermore, the excellent wettability of this hybrid SAM is highly conducive to the fabrication of large-area devices. A certified PCE of 22.74% has been achieved by a mini-module with an aperture area of 11.1 cm^2^, which demonstrates the potential of the buried bottom interface hybridization strategy for large-area PSM. Hou et al. investigated the surface packing and crystallization mechanisms of SAMs on TCO substrates and compared the impact of crystalline SAMs (c-SAMs) and amorphous SAMs (a-SAMs) on perovskite growth [67]. They observed that the perovskite film deposited on a-SAM exhibited a photoluminescence intensity that was tenfold higher and featured a narrower distribution compared to the perovskite film on c-SAM (Fig. 2 d). Additionally, the perovskite film on a-SAM demonstrated an average quasi-Fermi level splitting of 1.18 V, surpassing the value of 1.15 V observed for the film on c-SAM (Fig. 2 e). The results indicate that a-SAMs are more conducive to obtaining phase-uniform perovskite films, which is crucial for the scalable fabrication of PSCs. Based on a-SAMs, they achieved a PCE of 25.20% (certified at 24.35%) on devices with aperture area of 1 cm^2^.

**Fig. 3:**
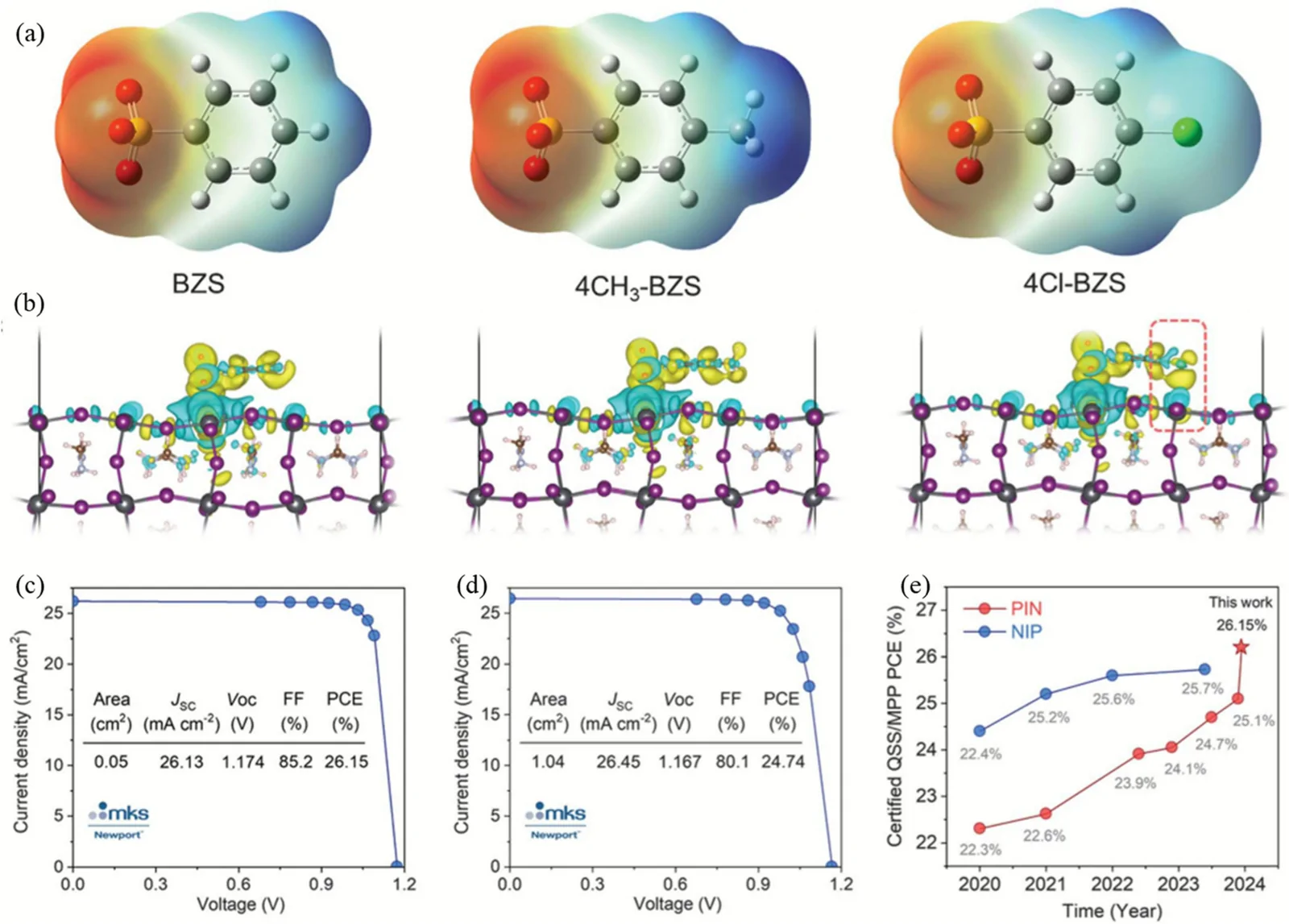
**a** Schematic illustrations and electrostatic potential of the BZS, 4CH_3_-BZS, and 4Cl-BZS ligands. **b** Atomic structures of ligand adsorbed in a planar or parallel orientation (Conf-para) on the perovskite surface. **c**–**d** *J*–*V* curves of champion devices with different areas: **c** 0.05-cm^2^ and **d** 1.04-cm^2^. **e** Summary of published nip and pin PSC performances in recent years. Reproduced with permission from Ref. [28]. Copyright 2024, AAAS

Ammonium ligands have been extensively utilized to passivate surface defects within perovskite layers. However, these linear-chain ligands bind exclusively to a single active binding site within the perovskite film, which creates a dense stacking layer oriented perpendicular to the perovskite surface. This configuration introduces an undesirable resistive barrier between the perovskite and the CTL [79]. To address this issue, Sargent et al. employed benzenesulfonate (BZS) ligands and their derivatives as passivating agents (Fig. 3 a) [28]. These molecules feature a flat benzene ring structure, which, upon binding to perovskites, is expected to align parallel to the perovskite surface. Furthermore, their research revealed that 4-chlorobenzenesulfonate (4Cl-BZS), characterized by the presence of chloride atom in the para position opposite to the sulfonate functional group, demonstrates the unique ability to simultaneously bind to two defect sites (Fig. 3 b). Thanks to the improved top interface achieved by 4Cl-BZS, devices with areas of 0.05 and 1.04 cm^2^ achieved certified PCEs of 26.15% and 24.74%, respectively (Fig. 3 c, d). It is noteworthy that this marks the first time that the certified PCE of inverted PSCs surpassed that of their regular counterparts (Fig. 3 e).

**Fig. 4:**
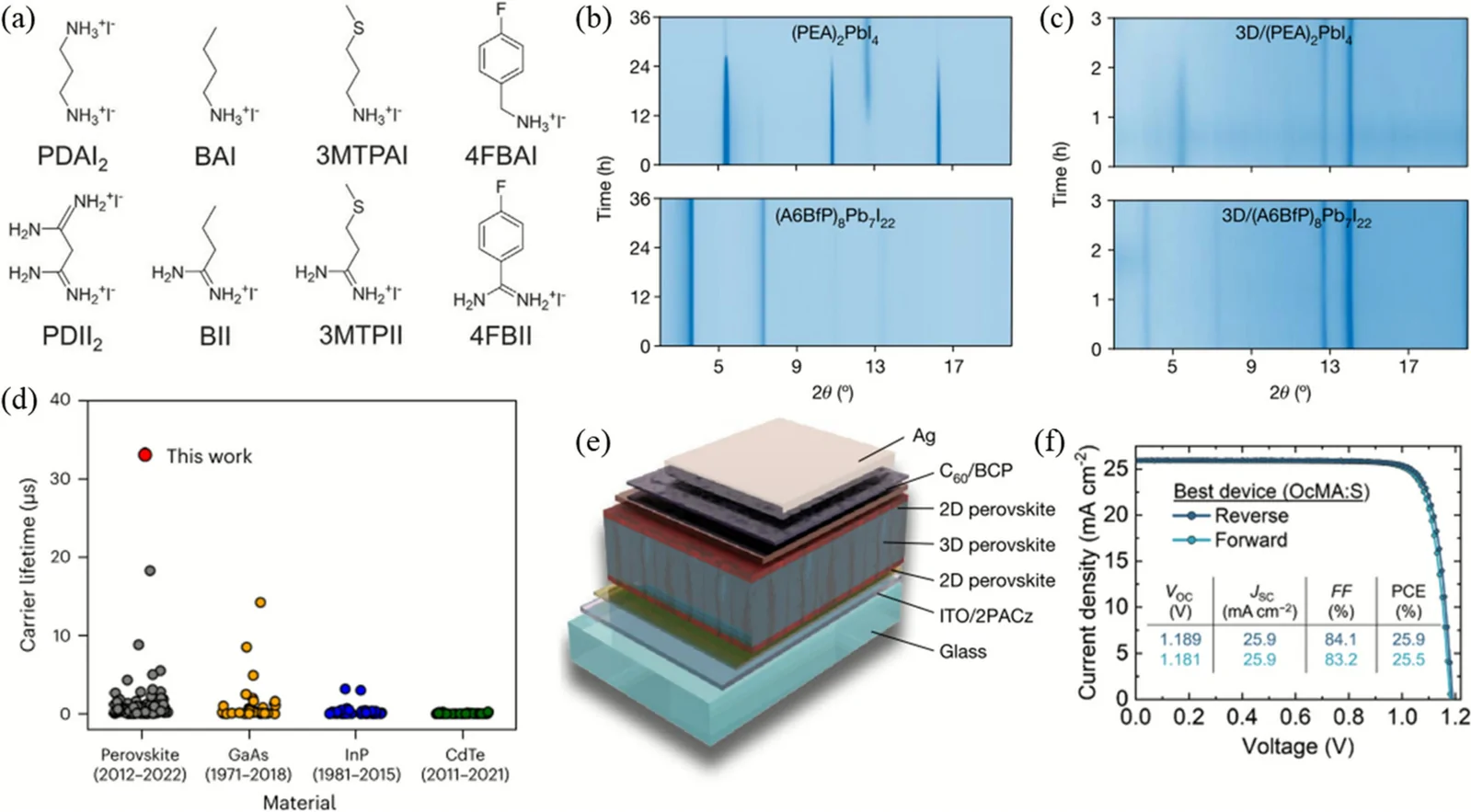
**a** Molecular structures of diverse ligands. Reproduced with permission from Ref. [56]. Copyright 2024, Springer Nature. **b** Time-dependent X-ray diffraction patterns of (PEA)_2_PbI_4_ and (A6BfP)_8_ Pb_7_ I_22_ films under 85% RH and 85 °C. **c** Time-dependent X-ray diffraction of 3D perovskite films with passivation of 2D (PEA)_2_ and 2D (A6BfP)_8_Pb_7_I_22_ under 85% RH and 85 °C. Reproduced with permission from Ref. [37]. Copyright 2024, Springer Nature. **d** Charge carrier lifetimes of various direct bandgap semiconducting materials reported over the past 50 years. Reproduced with permission from Ref. [38]. Copyright 2024, Springer Nature. **e** Schematic illustration of the device structure for PSCs featuring double-sided 2D/3D heterojunctions. Reproduced with permission from Ref. [82]. Copyright 2024, Springer Nature. **f** *J*–*V* curves of the device. Reproduced with permission from Ref. [39]. Copyright 2025, AAAS

Additionally, the same research group discovered that ammonium ligands undergo deprotonation under light and thermal stress. In response, they developed a library of amidinium ligands (Fig. 4 a), which are of interest due to their resonance-enhanced N–H bonds that may resist deprotonation [56]. This strategy enhances the thermal stability of passivation layers on perovskite surfaces, resulting in a more than tenfold reduction in the ligand deprotonation equilibrium constant. After illumination aging at 85 °C, the retention rate of photoluminescence quantum yield is doubled. Snaith et al. introduced a vapor-based amino-silane passivation method, which reduced the *V*_OC_ losses of PSCs with bandgaps ranging from 1.6–1.8 eV to approximately 100 mV (> 90% of the thermodynamic limit) [80]. Their findings revealed that primary, secondary, or tertiary amine-silanes individually exerted negligible or detrimental effects on perovskite crystallinity and charge transport. However, the combination of amino-silanes featuring both primary and secondary amines results in a 60-fold enhancement in photoluminescence quantum yield, while maintaining long-range conduction.

The formation of a 2D/3D heterojunction by growing a layer of 2D perovskite on the surface of 3D perovskite represents another prevalent passivation strategy. However, under thermal driving forces, the active cations within the 2D perovskite tend to migrate between the 2D and 3D perovskite layers [81]. This migration results in the disruption of the fragile corner-sharing octahedral connections within the perovskite structure, thereby hampering further advancements in device PCE and stability. Kanatzidis et al. introduced an interface passivation strategy based on 2D “perovskitoid” materials. In this approach, perovskitoid materials that exhibit simultaneous corner-, edge-, and face-sharing characteristics are employed to construct the interface passivation layer [37]. They explored a range of perovskitoids of varying dimensionality and discovered that cation migration within perovskitoid/3D perovskite heterostructures was suppressed compared to the 2D/3D perovskite scenario. Among the developed perovskitoid materials, the 2D (A6BfP)_8_Pb_7_I_22_ stands out for its exceptional performance. Notably, both the 2D (A6BfP)_8_Pb_7_I_22_ and 3D perovskite films passivated with 2D (A6BfP)_8_Pb_7_I_22_ exhibit prolonged structural stability under conditions of 85% RH and 85 °C, as confirmed by the results obtained from time-dependent X-ray diffraction patterns (Fig. 4 b, c). PSCs based on this “perovskitoid/3D perovskite heterojunction” achieved a certified PCE of 24.6% on a square centimeter scale. Bulović et al. found that treatment with hexylammonium bromide results in the simultaneous formation of an iodide-rich 2D layer accompanied by a bromide halide gradient that extends from defective surfaces and grain boundaries into the bulk 3D layer [38]. This strategy reduces the interface recombination velocity to less than 7 cm s^− 1^ and enables carrier lifetimes to be extended to over 30 μs, which represents the longest reported value for a direct bandgap semiconductor (Fig. 4 d). De Wolf et al. demonstrated that long alkylamine ligands can generate near-phase-pure 2D perovskite on both the top and bottom of 3D perovskite [82]. Building upon this discovery, they successfully fabricated inverted PSCs incorporating double-sided 2D/3D heterojunctions (Fig. 4 e). By leveraging the regulatory effects of 2D perovskite on charge recombination, ion migration, and electric field inhomogeneities at the interfaces, the double-sided 2D/3D heterojunction device yielded a stabilized PCE of 25.1%. Notably, the *V*_OC_ × FF value of this device stands at ~ 91% of the Shockley–Queisser limit, which represents one of the highest values reported among PSCs with only top-side or bulk passivation. Bawendi et al. discovered that the 2D/3D perovskite stack within a device undergoes dynamic evolution during its end-of-life decomposition process [39]. To address this issue, they employed a mixed-solvent approach to regulate the crystallinity and phase purity of the 2D intermediate layers. The resulting 2D/3D device achieved a PCE of 25.9% and exhibited excellent durability (Fig. 4 f). After being subjected to MPPT at 85 °C for 1074 h, it still retained 91% of its initial PCE.

### Charge Transport Layer Design

**Fig. 5:**
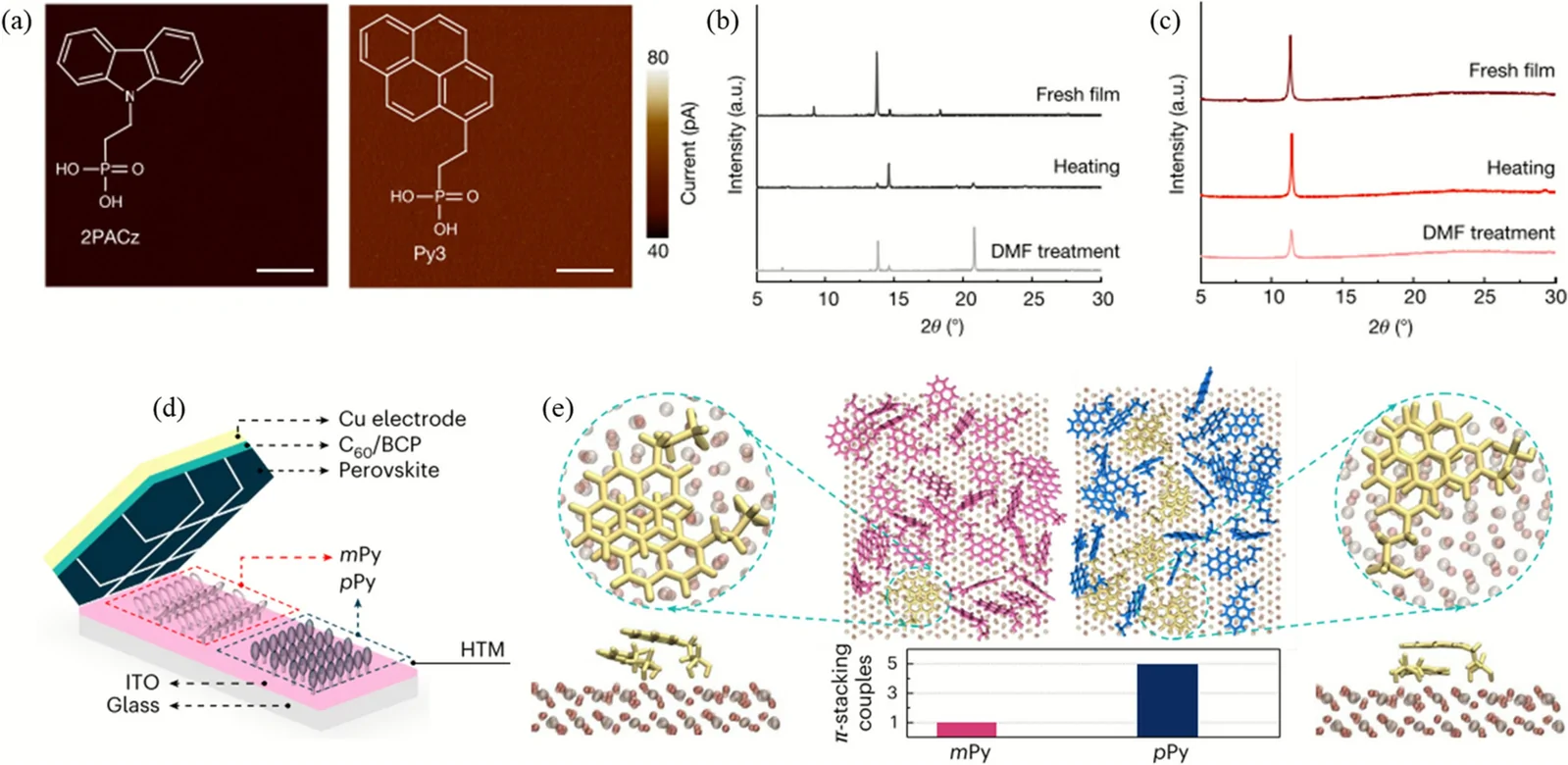
**a** Molecular structures of 2PACz and Py3, along with conductive atomic force microscopy images of ITO substrates coated with 2PACz and Py3. Scale bars, 2 μm. X-ray diffraction patterns of **b** 2PACz and **c** Py3 films before and after undergoing thermal treatment and organic solvent processing. Reproduced with permission from Ref. [64]. Copyright 2024, Springer Nature. **d** Device architecture and ideal spatial configuration of *m*Py and *p*Py molecules. **e** Top and side views of equilibrated molecular representations of *m*Py and *p*Py bonding on an ITO surface. Reproduced with permission from Ref. [85]. Copyright 2025, Springer Nature

To ensure efficient charge transport, commonly utilized SAMs often rely on π-conjugated structures substituted with heteroatoms such as nitrogen, sulfur, oxygen, and others [83]. However, under external bias or illumination conditions, such structures exhibit instability due to the polarized chemical bonds [84]. To establish a robust interfacial contact, Xue et al. designed a molecular structure, (2-(pyren-1-yl)ethyl)phosphonic acid, termed Py3 [64]. Py3 is distinguished by its pyrene conjugation core devoid of any heteroatom substitution, while exhibiting exceptional electronic properties at the interface (Fig. 5 a). Compared with conventional 2PACz, its *peri*-fused polyaromatic structure is chemically inert and conformationally rigid, which endow it with structural stability even after exposure to heat and treatment with DMF (Fig. 5 b, c). Devices utilizing Py3 as the HTL achieved a PCE of 26.1% (certified 25.7%) and demonstrated excellent operational stability in various accelerated aging tests. Xu et al. successfully synthesized an axially symmetric molecule, (2-(pyren-2-yl)ethyl)phosphonic acid (*p*Py), which features uniform electron delocalization. Compared to asymmetric *m*Py molecules, this symmetric structure exhibits the ability to form long-range ordered *π*–*π* stacking assemblies on ITO. (Fig. 5 d, e) [85]. Furthermore, the *p*Py thin film exhibits a strong and well-defined Debye–Scherrer ring at q = 0.27 Å⁻^1^, demonstrating a highly ordered face-up orientation and a more uniform spatial distribution. These characteristics effectively facilitate charge transport. PSCs fabricated using *p*Py achieved a remarkable PCE of 26.6% and maintained 94% of their initial PCE after being subjected to continuous simulated solar illumination for 3000 h under the ISOS-L-1I protocol. Although highly ordered SAMs generally facilitate the transport of charge carriers, structural deformation and phase transitions induced by external stress can disrupt this orderliness, thereby constraining the long-term operational stability of PSCs. Wang et al. demonstrated a molecular contact layer featuring an orthogonal π-skeleton [86]. This molecular design results in a disordered amorphous structure that is not only highly stable but also exhibits exceptional charge selectivity and transport capabilities. Zhu et al. achieved co-deposition of a novel p-type small molecule (D4PA) with perovskite thin films [26]. The C–C coupling within the D4PA molecule is capable to form strong multi-anchoring interactions with both the perovskite and the substrate. This interaction not only enhances interfacial charge transport but also suppresses the formation of defects in the perovskite layer. Moreover, the steric hindrance introduced by the C–C coupling leads to a distorted molecular conformation, which effectively prevents molecular aggregation. As a result, D4PA-based devices demonstrated a PCE of 26.83% (certified 26.72%) and a certified MPPT PCE of 26.14%. After continuous operation at the maximum power point for 2500 h, the devices still retained over 97.2% of their initial PCE. Brabec et al. demonstrate a closed-loop workflow grounded in molecular descriptors [66]. This ML model integrates Bayesian optimization with high-throughput synthesis of organic semiconductors to generate extensive datasets, aiming to discover novel hole-transporting materials with tailored properties suitable for photovoltaic applications. They have obtained a series of high-performance molecules, and on the basis of these molecules, they achieved a PCE of 26.2% (certified 25.9%) in PSCs.

**Fig. 6:**
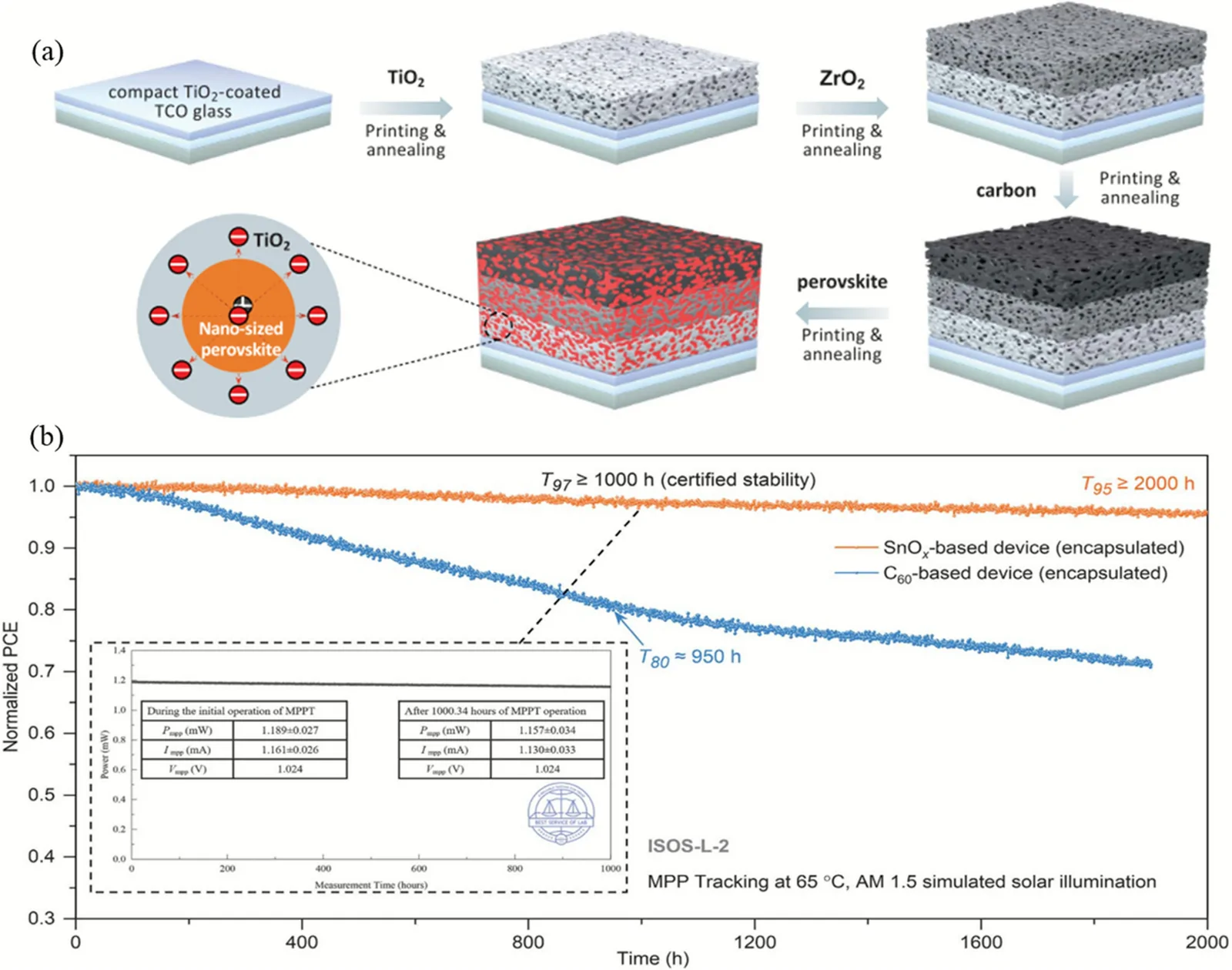
**a** Schematics for the fully wet-processed fabrication process of p-MPSCs and the charge separation process in the printed triple-layer mesoscopic structure. Reproduced with permission from Ref. [88]. Copyright 2024, AAAS. **b** Operational stability of devices at temperature of 65 °C. Reproduced with permission from Ref. [92]. Copyright 2024, AAAS

Printable mesoscopic PSCs (p-MPSCs) eliminate the necessity for the additional HTL required in conventional p-n junctions, yet they demonstrated relatively lower PCEs of ~ 19% [87]. Han et al. devised a three-layered mesoporous structure comprising semiconducting titanium dioxide (TiO_2_), insulating zirconium dioxide (ZrO_2_), and conductive carbon [88]. The perovskite material was infiltrated within the 3D interconnected pores and contacted with both the TiO_2_ and carbon mesoporous layers (Fig. 6 a). This strategy led to the fabrication of p-MPSCs achieving a remarkable PCE of up to 22.2%. Employing ETLs with a porous structure augments the contact area with the perovskite layer, thereby facilitating charge separation and extraction. TiO_2_ is the most frequently utilized mesoporous electron transport material. However, its requirement for sintering at temperatures exceeding 500 °C and its susceptibility to photocatalytic reactions under illumination pose limitations on the stability of the PSCs [89]. To address this issue, Park et al. utilized molybdenum disulfide (MoS_2_) as an alternative to TiO_2_ as the ETL in the fabrication of PSCs [90]. This strategy achieved PCEs of 25.7% (certified at 25.4%) and 22.4% for PSCs with aperture areas of 0.08 and 1 cm^2^, respectively. Moreover, MoS_2_-based PSCs exhibit exceptional photostability, capable of maintaining stable operation for over 2000 h under continuous illumination. Tin dioxide (SnO_2_) ETLs prepared via chemical bath deposition (CBD) are crucial for achieving high PCE in PSCs. Nonetheless, CBD is a time-consuming process. To address this issue, Seok et al. proposed a novel method for synthesizing SnO_2_ colloids in an H_2_O_2_ solution [91]. This approach resulted in ultrafine SnO_2_ colloids with a particle size of 4–6 nm, which exhibited reduced oxygen vacancies and a uniform dispersion. Moreover, they discovered that sonication, coupled with the addition of formamidinium chloride (FACl), facilitates the formation of defect-free interfaces with perovskites. Utilizing SnO_2_-FACl as the ETL enabled the realization of high-performance PSCs with a high PCE of 26.05% (certified at 25.54%). Zhu et al. grew a layer of SnO_x_ on the top surface of the perovskite via ALD to replace fullerene, serving as the ETL in inverted PSCs [92]. By manipulating the oxygen vacancy defects within the SnO_x_ layer, the researchers achieved PCEs > 25%. Furthermore, the device boasts *T*_97_ and *T*_95_ lifetimes exceeding 1000 and 2000 h, respectively (Fig. 6 b).

### Perovskite Crystallization

The crystallization quality of perovskite films is pivotal for determining the photovoltaic performance of PSCs. High-quality perovskite thin films with large grain sizes, low-defect densities, and uniform morphology facilitate efficient charge carrier transport by reducing recombination losses and extending carrier lifetimes. Key factors influencing crystallization include precursor composition, solvent volatility, annealing protocols, and environmental conditions during deposition. FAPbI₃ stands out as a leading candidate for high-efficiency PSCs due to its narrow bandgap (~ 1.48 eV) and superior thermal stability compared to MAPbI₃. However, its α-phase (black phase) is metastable at room temperature and readily converts to the non-photactive δ-phase (yellow phase), limiting practical application [93]. Challenges persist in balancing phase stability, scalability, and environmental robustness, particularly under humid or thermal stress.

**Fig. 7:**
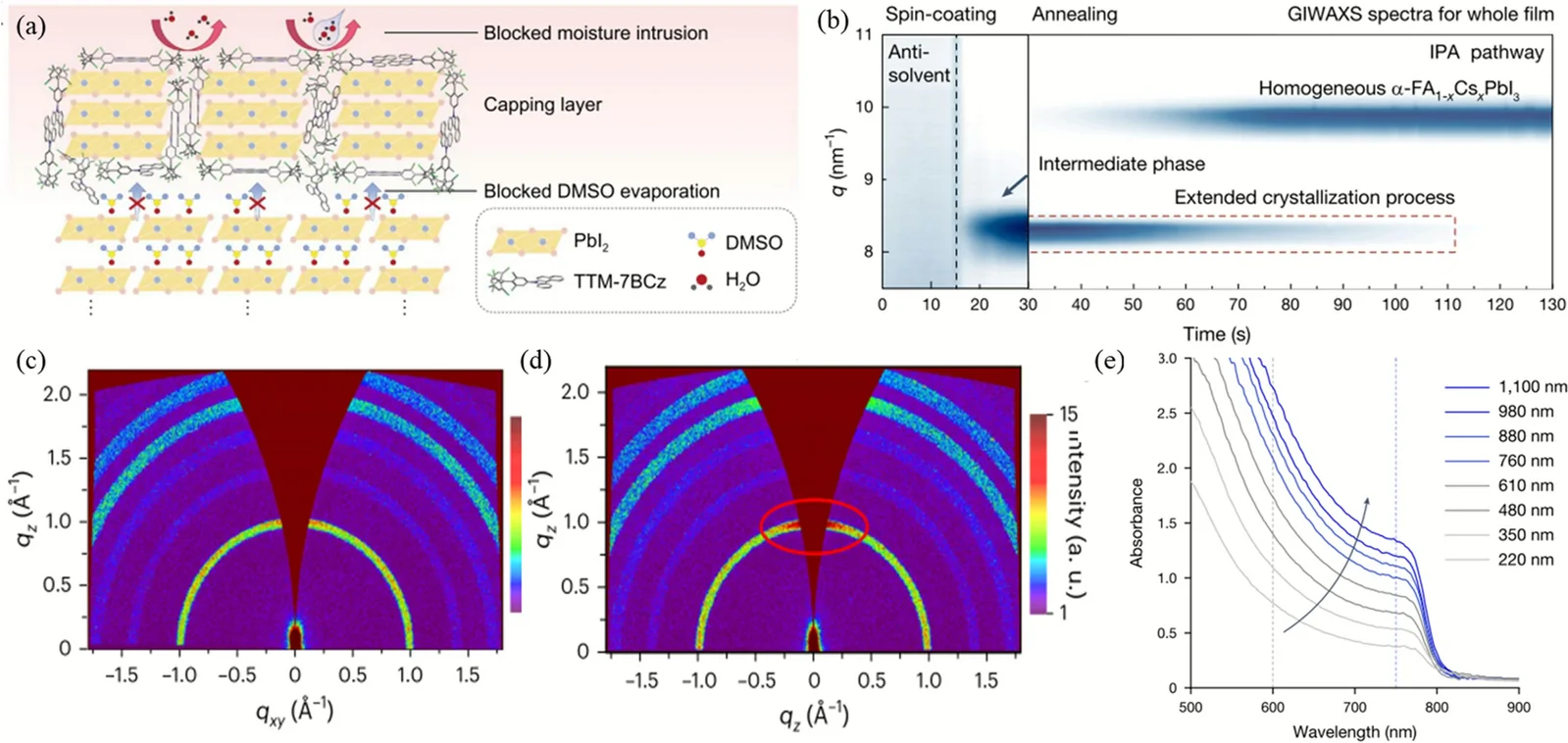
**a** Schematic representation and functional description of intermediate films with an in situ–formed capping layer. Reproduced with permission from Ref. [101]. Copyright 2024, AAAS. **b** Time-resolved grazing incidence wide-angle X-ray scattering spectra for IPA α-FA_1−x_CsxPbI_3_ films. Reproduced with permission from Ref. [55]. Copyright 2024, Springer Nature. GIWAXS patterns of **c** control and **d** target perovskite films. The enhanced intensity of the (100) diffraction ring near the 90° azimuthal angle is highlighted by the red ellipse. Reproduced with permission from Ref. [58]. Copyright 2025, Springer Nature. **e** UV–vis absorbance spectra of FA-based perovskite absorbers with varying thicknesses. Reproduced with permission from Ref. [104]. Copyright 2024, Springer Nature

Lewis bases are of paramount importance in the formation of the α-FAPbI₃ phase. Nevertheless, their roles present a paradox—strong binding is essential for stabilizing the intermediate δ phase, whereas weak binding is required for swift removal to promote phase transition and grain growth. To tackle this dilemma, Yan et al. introduced a strategy of “on-demand generation of Lewis base molecules” [94]. This approach optimizes the crystallization of α-FAPbI₃ thin films, achieving a homogeneous distribution of A-site cations, larger grain sizes, and a reduced number of interfacial defects. The tolerance factor of α-FAPbI_3_ is 0.987, sitting on the edge of the stability range (0.8–1.0) for perovskite structures [95]. During crystallization, FAPbI_3_ readily forms a non-photovoltaic hexagonal phase with face-shared octahedra, which leads to decreases in both the PCE and stability of PSCs. Choy et al. reported the utilization of a hydrogen-bonded eutectic molecule (EM) as a [PbI_6_]^4−^ octahedral ligand to foster the predominant formation of corner-sharing octahedra [96]. The elevated ratio of corner-sharing to face-sharing octahedra can catalyze a thorough phase transformation and facilitate the development of a pure cubic perovskite structure. Similarly, Zhou et al. developed a kinetic modulation strategy that utilizes the co-generated volatile iodine intercalation and deintercalation processes to assist in the preparation of high-quality and stable non-alloyed α-FAPbI_3_ thin films [97]. Mohite et al. created lattice-matched 2D perovskites as growth templates [98]. When pure FAPbI_3_ precursor solutions come into contacting with these 2D perovskites, the black phase preferentially forms at 100 °C, significantly lower than the standard annealing temperature of 150 °C for FAPbI_3_. During this process, 2D perovskites containing FA function as the cage cation, and the bulk FAPbI₃ films experience a slight compression to align with the (011) interplanar distances of the 2D perovskite seed. In addition to composition, the conventional hygroscopic solvent dimethyl sulfoxide (DMSO) also contributes to the formation of δ-FAPbI_3_ under high RH conditions [99, 100]. Particularly in humid air, the detrimental hydration effect induced by DMSO outweighs its benefits in facilitating intermolecular exchange. This situation restricts the preparation of perovskite thin films to inert atmospheres. To address this issue, Xiao et al. utilized chlorine-containing organic molecules to form an in situ protective layer during the crystallization and shield the perovskite from moisture infiltration (Fig. 7 a) [101]. This innovative strategy preserved the beneficial attributes of DMSO-PbI_2_ coordination and facilitated the fabrication of PSCs at a RH of 80%. The as-prepared device achieved a PCE of 23.4% and exhibits excellent operational stability in air environments. Notably, all state-of-the-art α-FAPbI_3_-based PSCs reported thus far incorporate methylammonium chloride (MACl) as an additive to stabilize and promote the metastable α-FAPbI_3_ phase [102]. However, the volatilization of MACl gives rise to organic residues (specifically, MA), which constrains the stability of the device at elevated temperatures. The Yuan team presented an intermediate phase-assisted (IPA) crystallization pathway facilitated by acetate surface ligation [55]. Here, acetate anions, which possess a strong surface binding affinity, are capable to reduce the relative formation energy of the intermediate δ phase. Consequently, this switches the crystallization pathway from a single-step process to an IPA one. This approach extends the crystallization process, thereby enabling the production of homogeneous α-FACsPbI_3_ films without the need for MA additives (Fig. 7 b). As a result, they elevated the certified PCE of α-phase FA_1-x_Cs_x_PbI_3_ PSCs without MACl from ~ 24% to 25.94%. Chen et al. regulated the nucleation and growth of FACs-based perovskite through the aromatic interactions between naphthylammonium salts and naphthalenesulfonates. The ammonium group of the naphthylammonium salts occupied the formamidinium sites, while the sulfonate group of the naphthalenesulfonates coordinated with lead ions. These interactions facilitated ordered epitaxial crystallization along the (100) plane, enhancing defect passivation and carrier transport (Fig. 7 c, d). Ultimately, the researchers achieved a PCE of 27.02% (certified 26.88%). After encapsulation, the device retained 98.2% of its initial PCE after undergoing 2000 h of MPPT under continuous illumination in ambient air [58].

The thickness of the active layer in inverted PSCs is generally less than 500 nm, which leads to inadequate absorption of light waves, particularly in the long-wavelength region (Fig. 7 e). Consequently, the short-circuit current density (*J*_SC_) of inverted PSCs is typically lower than that of their regular counterparts [103]. Zhu et al. achieved the formation of coherent grain boundaries by facilitating the growth of grains with high Miller index orientations on those with low Miller index orientations in a stable atmosphere [104]. This approach yielded high-quality formamidinium-based perovskite films with micrometer-scale thickness. Benefiting from the sufficient absorption of light waves by this thick responsive layer, the cell PCE reached 26.1%, accompanied by a high *J*_SC_ of 26.3 mA cm^− 2^. Xue et al. reported a series of high-entropy organic–inorganic hybrid perovskites for photovoltaic applications by mixing different A-site organic cations with varying alkyl chains [65]. The entropy increase originates from the hybrid crystal structure combining an ordered inorganic framework with a disordered organic component. Compared to their single-component counterparts, high-entropy mixed perovskites exhibit superior properties, including enhanced structural transition and thermal stress recovery capabilities. Tin (Sn)-based PSCs (TPSCs) are regarded as one of the best options for lead (Pb)-free photovoltaic technology due to their low toxicity and high theoretical PCE [105]. However, due to poor control over perovskite film growth and the inherent susceptibility of Sn^2^⁺ to oxidation, the PCE and stability of TPSCs lag significantly behind their Pb-based counterparts. Wei et al. synthesized two pyridyl-substituted fulleropyrrolidines (PPF) with cis (CPPF) and trans (TPPF) configurations and utilized them as precursor additives [106]. The spatial configurations of CPPF and TPPF impact the electron density distribution and interactions with perovskite components. Compared to CPPF, TPPF features spatially separated pyridyl groups that can capture more perovskite colloids through coordination bonds. TPPF also resides at grain boundaries, which enhances the alignment of interface energy levels and mitigates Sn^2^⁺ oxidation. Consequently, TPPF-based TPSCs demonstrate a high PCE of 15.38% (certified at 15.14%) and exceptional stability. Constructing 2D/3D heterostructures offers an effective means to regulate the crystallization process and suppress defect formation, thereby enabling the preparation of high-quality Sn-based perovskite films. However, the high aggregation barrier impedes the formation of stable clusters by large-sized 2D perovskite colloids, resulting in slower nucleation kinetics of 2D Sn-based perovskites compared to their 3D counterparts. Wang et al. reduced the colloid size to lower the aggregation barrier by introducing small-sized Cs⁺ to partially replace the bulky organic cations in the 2D materials [107]. This strategy facilitated the co-aggregation of 2D and 3D Sn-based perovskite colloids in the precursor solution, achieving synchronization of nucleation kinetics. Based on this, TPSCs attained an outstanding PCE of 17.13% (certified 16.65%).

### Flexible Device

The FPSCs have garnered considerable attention due to their flexibility and high power-to-weight compatibility [108–110]. However, the poor interfacial adhesion and significant deformation of flexible substrates lead to poor perovskite crystallization and interfacial contact, which hinders the performance of FPSCs [111, 112]. Ge et al. incorporated a zwitterion elastomer (SBMA) into the perovskite film, forming an intermediate SBMA-PbI_2_ adduct through in situ cross-linking to regulate the nucleation and crystallization of perovskite [70]. The cross-linked elastomer located at the grain boundaries imparted instant self-healing capabilities to the flexible perovskite film under mild processing conditions (40 °C for 15 min). The resulting device achieved a PCE of 24.51% (certified at 24.04%) and exhibited exceptional mechanical stability and durability. After 10,000 bending cycles, over 90% of the initial PCE was maintained. Song et al. designed a self-healing ionic conductive elastomer (ICE), which incorporated imidazolium-based ionic liquids, and integrated it into perovskite films [113]. This innovation enabled real-time self-repair of grain boundary cracks in flexible PSCs at ~ 25 °C, as well as modulation of their electrical properties. Consequently, the device achieved a high PCE of 24.84%. To address the issue of acid sensitivity in flexible ITO substrates, Yi et al. utilized SnSO_4_ as a tin source instead of the conventional SnCl_2_ [53]. This approach enables the controlled growth of SnO_2_ thin films under constant pH conditions without the need for strong acids. The resulting SnO_2_ particles exhibit uniformity and compactness, with high coverage and reproducibility. FPSCs based on this method achieved a PCE of up to 25.09% (certified at 24.90%), which is the highest PCE record reported to date for FPSCs.

**Fig. 8:**
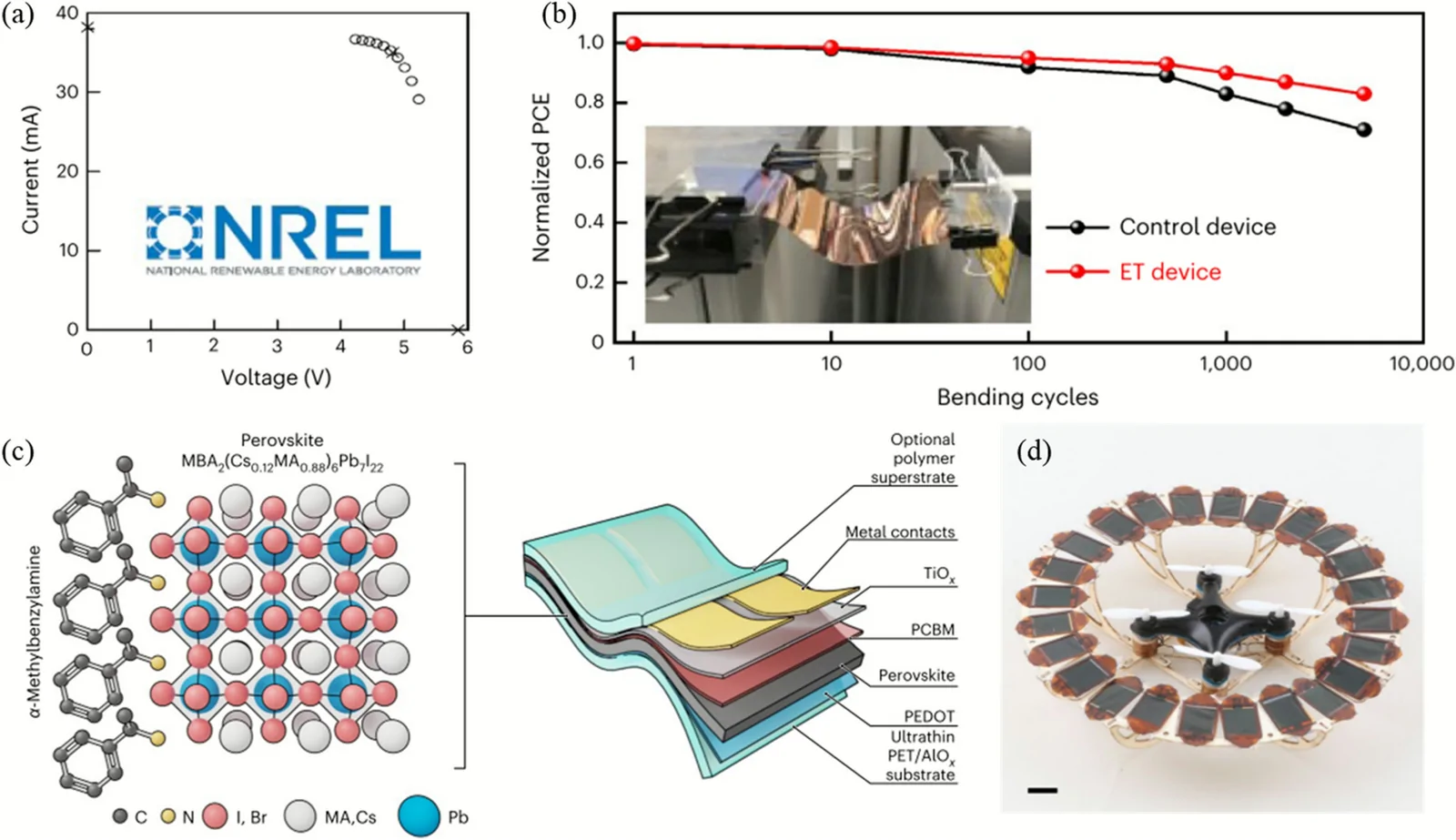
**a** Certification of stabilized PV performance for a minimodule by National Renewable Energy Laboratory (NREL). **b** Normalized PCEs of the control and ET devices as a function of bending cycles. Reproduced with permission from Ref. [71]. Copyright 2024, Springer Nature. **c** Schematic illustration of the MBAI and corresponding device architecture. **d** Photo of the assembled solar-powered quadrotor (scale bar, 1 cm). Reproduced with permission from Ref. [54]. Copyright 2024, Springer Nature

To enhance the contact between perovskite and flexible substrates, Huang et al. incorporated the organic molecule entinostat (ET) into the HTL of inverted FPSCs [71]. By virtue of the interaction between ET and the perovskite, they managed to enhance the adhesion between the perovskite and the ITO substrate, while simultaneously minimizing void formation at the bottom interface. The fabricated FPSCs exhibited PCEs of 23.4% for small-area devices and 20.1% (certified at 19.0%) for minimodules (Fig. 8 a). Encouragingly, after undergoing 5000 bending cycles, the flexible minimodule retained 84% of its initial PCE (Fig. 8 b). Kaltenbrunner et al. fabricated devices directly on ultra-thin polymer foil coated with an alumina barrier layer to ensure environmental and mechanical stability without compromising weight and flexibility [54]. By introducing α-methylbenzyl ammonium iodide (MBAI) into the perovskite absorber layer (Fig. 8 c), they demonstrated a champion power per weight of 44 W g⁻^1^ (average: 41 W g⁻^1^), an *V*_OC_ of 1.15 V, and a champion PCE of 20.1% (average: 18.1%). To showcase scalability, a photovoltaic module consisting of 24 interconnected 1 cm^2^ solar cells was fabricated and demonstrated energy autonomy for a hybrid solar-powered quadrotor. Surprisingly, the photovoltaic energy system accounts for a mere 1/400 of the weight of the designed drone (Fig. 8 d).

## Perovskite-Based Tandem Solar Cells

Due to the diverse range of bandgap options available in perovskite materials, PSCs can be integrated with other types of solar cells to form tandem solar cells [15, 114]. Typical combinations include perovskite/silicon, perovskite/perovskite, and perovskite/organic. In perovskite-based tandem solar cells, wide bandgap (WBG) perovskites are utilized as the top cell, while narrow bandgap (NBG) perovskites or other materials serve as the bottom cell. Compared to single-junction PSCs, tandem solar cells have long been recognized as a promising avenue for capturing a broader spectrum of sunlight and achieving higher PCEs. Currently, the certified PCE of perovskite/silicon tandem solar cells has surpassed 34%, which exceeds the Shockley–Queisser limit for single-junction photovoltaic cells [52]. Meanwhile, significant strides have been made in the performance of perovskite/perovskite and perovskite/organic tandem cells over the past two years [115–117]. The field placed significant emphasis on the regulation of crystallization in WBG and NBG perovskites, as well as interface modification between perovskites and CTLs. Perovskite-based tandem solar cells with outstanding certified PCEs are summarized in Table 2.

**Table 2:** Summary of certified photovoltaic parameters of perovskite-based tandem solar cells

Device type	Area(cm^2^)	*V*_OC_ (V)	*J*_SC_ (mA cm^− 2^)	FF (%)	PCE (%)	Certified PCE (%) /Institution	References
Perovskite/Silicon	0.9972	1.925	20.713	78.9	31.67	31.46/SIMIT	[118]
Perovskite/Silicon	1	1.98	20.68	83.2	34.08	33.89/NREL	[29]
Perovskite/Silicon	1	1.98	20.51	83.4	33.86	33.59/SIMIT	[119]
Perovskite/Silicon	1	1.99	20.7	83.0	34.2	34.2/NREL	[120]
Perovskite/Silicon	1	1.942	20.4	81.27	32.5	32.2/NIM	[121]
Perovskite/Silicon	1.004	1.996	20.80	83.63	–	34.58/ESTI	[122]
Perovskite/Silicon	1.04	1.985	21.02	81.6	34	33.7/ESTI	[123]
Perovskite/Silicon	25	1.896	18.96	80.1	29.4	28.8/NIM	[121]
Perovskite/Perovskite	0.042	2.099	15.79	81.37	27.3	26.96/SIMIT	[124]
Perovskite/Perovskite	0.049	2.156	15.46	82.2	28.8	27.4/JET	[125]
Perovskite/Perovskite	0.0395	2.134	16.05	84.52	28.95	28.87/SIMIT	[126]
Perovskite/Perovskite	1.05	2.17	16.4	80.2	28.5	28.2/JET	[115]
Perovskite/Perovskite	20.25	17.26	1.86	77.5	24.9	24.5/JET	[47]
Perovskite/Organic	0.058	2.12	14.68	80.53	25.82	25.06/SIMIT	[127]
Perovskite/Organic	0.0419	2.145	14.2	79.67	25.22	24.27/SIMIT	[128]
Perovskite/Organic	0.09	2.157	15.303	77.82	26.4	25.7/IEE CAS	[116]
Perovskite/Organic	1.019	2.14	15.15	82.4	26.7	26.4/SIMIT	[129]

ESTI: European Solar Test Installation, Italy; JET: Japan Electrical Safety & Environment Technology Laboratories, Japan

### Perovskite/Silicon Tandem Cells

**Fig. 9:**
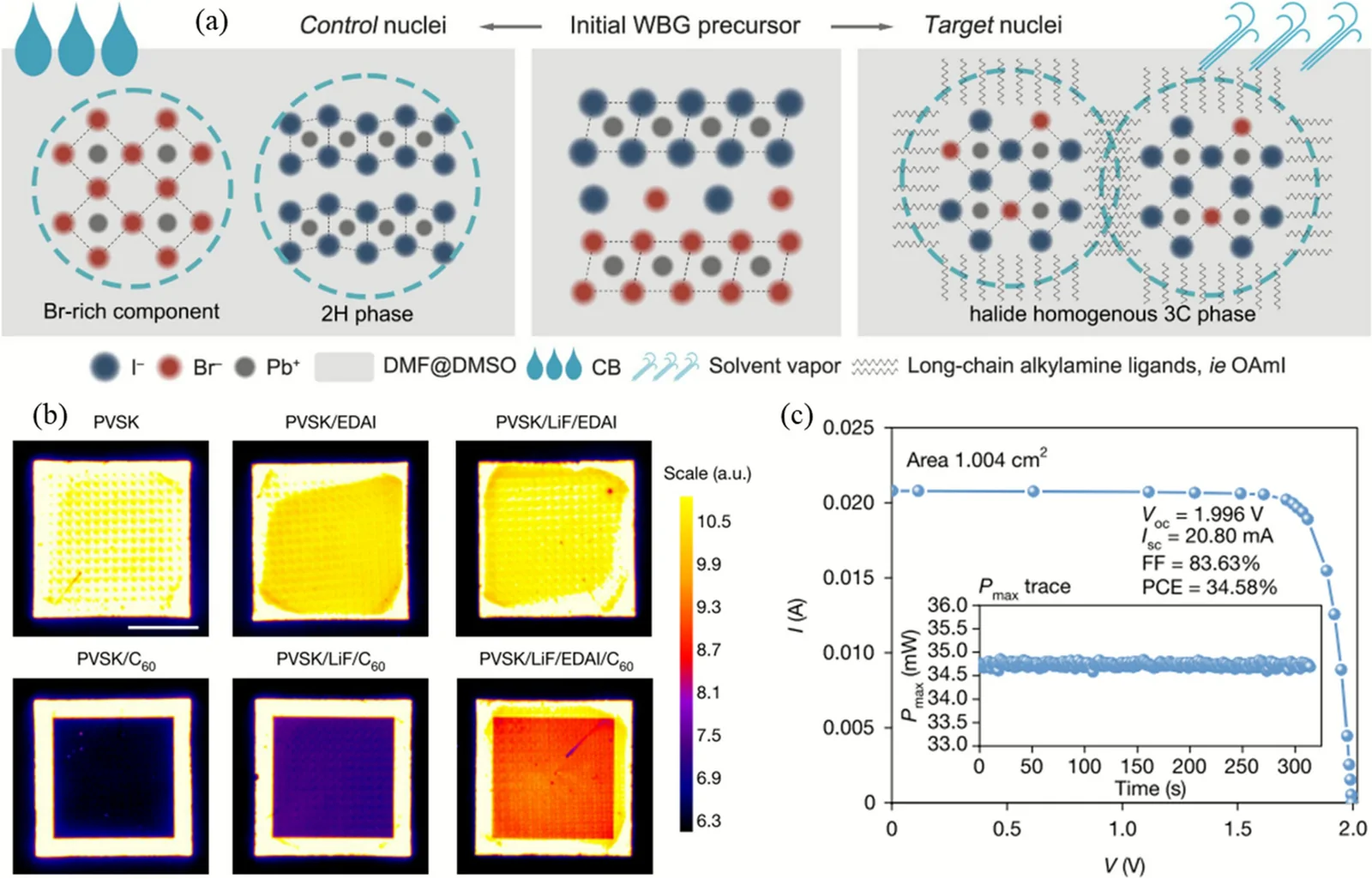
**a** Schematic illustration of nuclei engineering for various WBG absorbers. Reproduced with permission from Ref. [121]. Copyright 2024, AAAS. **b** Photoluminescence imaging spectra of different stacked surfaces. The substrate size is around 20 mm × 20 mm, and C_60_ deposition was defined by a mask of 15 mm × 15 mm. Scale bar, 10 mm. Reproduced with permission from Ref. [29]. Copyright 2024, Springer Nature. **c** Certified *I–V* curve of one HTL20-based tandem cell measured by the European Solar Test Installation. Reproduced with permission from Ref. [122]. Copyright 2025, Springer Nature

In perovskite/silicon tandem solar cells, the WBG perovskite serving as the top cell is prone to ion migration and phase separation, which undermines the long-term stability of the tandem cells [130]. Chen et al. introduced a straightforward and versatile strategy for controlling the crystallization of WBG perovskite through nucleus engineering [121]. This approach optimizes the texture of the WBG perovskite thin film by regulating the dominant nuclei in the precursor solution. Specifically, by nucleating the pesudocubic phase (labeled as 3C) prior to the formation of bromide-rich aggregates and the hexagonal phase (labeled as 2H), the growth of WBG films can be controlled to render it insensitive to composition variations (Fig. 9 a). The resultant perovskite/ silicon tandem cells achieved an PCE of 29.4% (certified at 28.8%) over an active area of 25 cm^2^ and 32.5% over 1 cm^2^. Additionally, De Wolf et al. incorporated methylenediammonium dichloride into the perovskite precursor solution, which facilitated the in situ formation of tetrahydrotriazinium (THTZ-H^+^) during the crystallization of the thin film [123]. The cyclic nature of the THTZ-H^+^ cation allows it to form hydrogen bonds with iodide in multiple orientations, leading to strong interactions with the lead octahedra within the perovskite lattice. This strategy addresses crystal defects and film inhomogeneity in WBG perovskites, which enhances their stability under prolonged illumination and thermal exposure. Monolithic perovskite/silicon tandem devices based on THTZ-H⁺ achieved a certified PCE of 33.7% for a 1 cm^2^ active area.

The inherent trade-off between passivation and charge extraction poses a constraint on the PCE improvement of perovskite/silicon tandem cells. Xu et al. overcame this drawback by introducing a thin lithium fluoride (LiF) layer, followed by the deposition of a short-chain ethylenediammonium diiodide (EDAI) molecule at the interface between the WBG perovskite and the ETL [29]. Compared to interfaces where C_60_ is directly deposited on the perovskite film, or where only LiF or EDAI serves as the passivation layer, the EDAI/LiF bilayer passivated interface exhibits the highest photoluminescence intensity and PLQY (Fig. 9 b). This strategy mitigates interfacial defects, thereby reducing the associated non-radiative recombination losses. As a result, the perovskite/silicon tandem cell achieved a certified stabilized PCE of 33.89%, accompanied by an impressive FF of 83.0% and an *V*_OC_ of nearly 1.97 V. This marks the first reported instance where the certified PCE of a dual-junction tandem solar cell surpasses the single-junction Shockley–Queisser limit of 33.7%. HTLs commonly utilized in perovskite top cells often encounter issues such as defects, non-conformal deposition, or dewetting of the overlying perovskite on textured silicon bottom cells [131]. To address this issue, He et al. devised an asymmetric SAM molecule named HTL201 [122]. This molecule features a carbazole core, with side chains incorporating anchoring groups and spacers, and it serves as the HTL in perovskite/silicon tandem solar cells. Compared to traditional symmetric SAM molecules, the HTL201 molecule enhances coverage and interfacial compatibility by minimizing steric hindrance and strengthening the interaction with the TCO. This strategy enables perovskite/silicon tandem solar cells to achieve a certified PCE of up to 34.58% with an impressive *V*_OC_ of nearly 2 V (Fig. 9 c). Qin et al. designed a diradical-based SAMs using a donor–acceptor conjugation strategy, which enhanced the hole-transporting performance [120]. This diradical-based SAMs exhibit outstanding photothermal and electrochemical stability, along with excellent uniformity and large-area solution processability. In addition, Yang et al. carried out the co-deposition of the perovskite precursor ink and copper(I) thiocyanate (CuSCN) onto the ITO recombination layer [118]. In this instance, CuSCN has been found to effectively passivate perovskite grain boundaries and exhibit highly efficient hole extraction, thereby facilitating the formation of localized hole-collecting contacts. The monolithic perovskite/silicon tandem device fabricated using this method achieved a certified PCE of 31.46% in a device with an aperture area of 1 cm^2^. Additionally, this method simultaneously enhanced the device's reproducibility, scalability, and humidity–heat stability.

### Perovskite/Perovskite Tandem Cells

**Fig. 10:**
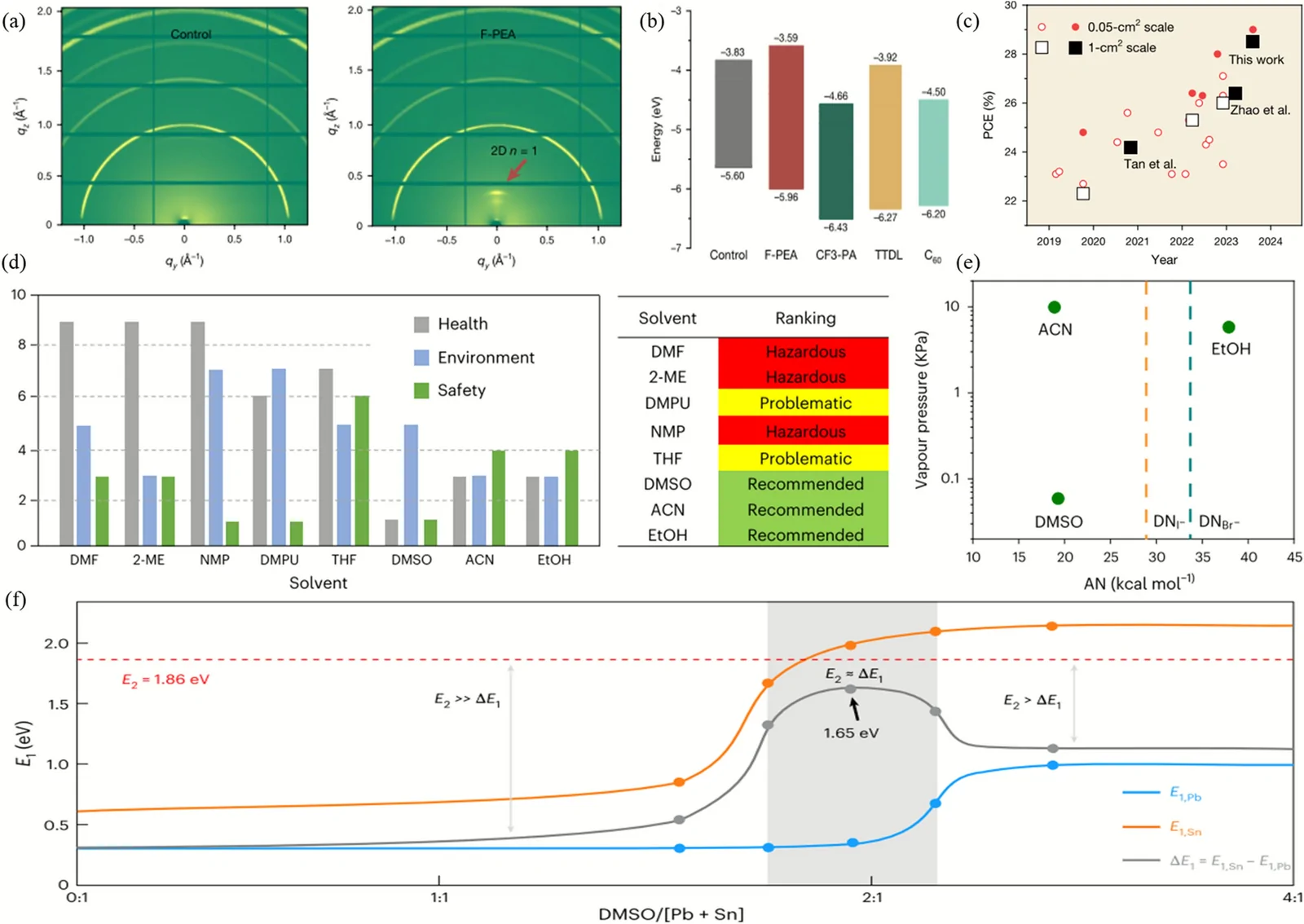
**a** Grazing incidence wide-angle X-ray scattering patterns of control, F-PEA and TTDL perovskite films. The color scale shows intensity in arbitrary units from low (dark green) to high (light green). **b** Band alignment of perovskite film treated by control, F-PEA, CF3-PA and TTDL. **c** Summary of the reported PCEs of all-perovskite tandem solar cells. Solid symbols are certified values. Reproduced with permission from Ref. [115]. Copyright 2024, Springer Nature. **d** Safety, health and environment impact of solvents outlined by CHEM21. **e** Vapor pressure and AN of the three studied solvents and DN of iodide and bromide ions. Reproduced with permission from Ref. [50]. Copyright 2024, Springer Nature. **f** Desorption barrier of Sn- and Pb-based perovskites as a function of DMSO/[Pb + Sn]. Reproduced with permission from Ref. [126]. Copyright 2025, Springer Nature

Due to the inhomogeneity of WBG PSCs, the certified PCE of all-perovskite tandem solar cells at the 1 cm^2^ scale lags behind that of their smaller counterparts [132]. Typically, the inhomogeneity of WBG perovskites is attributed to buried interfaces and the intrinsic crystallization of the perovskites themselves. Recently, Tan et al. discovered that the top interface, such as the deposited ETL (C_60_), is also a significant contributor to this inhomogeneity [115]. To address this issue, they introduced a mixture of 4-fluorophenethylamine (F-PEA) and 4-trifluoromethyl-phenylammonium (CF3-PA) at the top interface of the WBG perovskite to create a tailored 2D perovskite layer (TTDL). On the one hand, grazing incidence wide-angle X-ray scattering spectra revealed that F-PEA forms a 2D perovskite layer at the surface of the 3D perovskite, thereby mitigating contact losses and inhomogeneity (Fig. 10 a). On the other hand, the incorporation of CF3-PA bridges the energy level mismatch between F-PEA and C_60_, thereby enhancing charge extraction and transport (Fig. 10 b). The research team achieved a high *V*_OC_ of 1.35 V and a PCE of 20.5% in a WBG PSC with a bandgap of 1.77 eV. By stacking it with a NBG perovskite sub-cell, they constructed an all-perovskite tandem cell with an aperture area of 1.05 cm^2^. This cell achieved a PCE of 28.5% (certified at 28.2%), which was the highest PCE among its peers reported at that time (Fig. 10 c). Recently, Tan et al. employed 2D perovskite as an intermediate phase. By leveraging surface composition engineering, they facilitated heterogeneous nucleation along the (100) crystallographic plane direction, successfully achieving the preferred (100) crystal orientation [133]. Through this approach, the certified PCE of all-perovskite tandem solar cells reached 29.1%. Furthermore, the group conducted a comparison of the human and environmental-friendliness of various solvents (Fig. 10 d), upon which they developed a green solvent system for large-scale production of efficient WBG perovskites [50]. This system comprises DMSO and acetonitrile (ACN), which effectively dissolve the salts necessary for perovskite preparation. Additionally, the incorporation of ethyl alcohol (EtOH) serves to prevent precursor degradation, thereby extending the solution processing window. These synergistic modulations are attributed to the variations in vapor pressure, acceptor number (AN), and donor number (DN) of iodide and bromide ions exhibited by these three solvents (Fig. 10 e). Using this green solvent mixture, they successfully fabricated blade-coated WBG PSC with PCEs of 19.6% (for 1.78 eV) and 21.5% (for 1.68 eV), respectively. Subsequently, they successfully demonstrated a remarkable PCE of 23.8% for an all-perovskite tandem solar module with an aperture area of 20.25 cm^2^.

Sn–Pb mixed NBG perovskites are commonly employed as the bottom absorber layer in perovskite/perovskite tandem solar cells. However, Sn–Pb mixed perovskites suffer from an imbalanced crystallization process, which results in non-uniform thin films and subsequently degrades device performance. Tang et al. discovered that the crystallization of Sn-based perovskites is constrained by the desorption of DMSO, whereas Pb-based perovskites encounter a relatively minor DMSO desorption barrier (Fig. 10 f) [126]. By adjusting the DMSO content to regulate the reaction barriers in the mixed thin films, they achieved simultaneous Sn–Pb perovskite crystallization and the formation of high-quality, uniform thin films. Snaith conducted an in-depth investigation into the chemical properties of tin–lead perovskite precursor solutions [134]. The study revealed that Sn(II) species play a dominant role in interactions with precursors and additives. Furthermore, it has been unveiled carboxylic acids exhibit unique regulatory effects on the colloidal properties of solutions and the crystallization of thin films, whereas ammonium salts play a crucial role in enhancing the optoelectronic performance of the films. By combining these two functional groups, the resultant amino acid salt material notably enhances the semiconductor quality and uniformity of perovskite films. To suppress tin oxidation in tin–lead mixed perovskite materials, Ning et al. developed an electron-withdrawing chloromethyl phosphonate ligand based on substituent effects [124]. The incorporation of this electron-withdrawing ligand elevated the redox potential of tin adducts, resulting in a marked increase in the ionization potential of the perovskite structure. This enhancement facilitated more effective suppression of tin oxidation and reduced the defect density within tin-based perovskite films. Sargent et al. observed a composition gradient in tin–lead mixed perovskite films, with a higher tin content at the surface compared to the bulk [125]. To address this, they employed diamine compounds to chelate and subsequently remove tin atoms from the film surface. This approach achieved a more balanced Sn:Pb stoichiometric ratio and imparted resistance to tin oxidation at the film surface. The crystallization of tin-containing perovskite films is rapid, and the time window for large-area production is short, often leading to issues of uneven film formation. Tan's team extended the processing window for perovskite film formation and passivated the buried interfaces by incorporating a multifunctional zwitterionic buffer, glycine amide hydrochloride, into the precursor solution, enabling large-area and uniform fabrication of lead–tin perovskite films [47]. As a result, the steady-state PCE of the all-perovskite tandem module reached an impressive value of 24.5%.

### Perovskite/Organic Tandem Cells

**Fig. 11:**
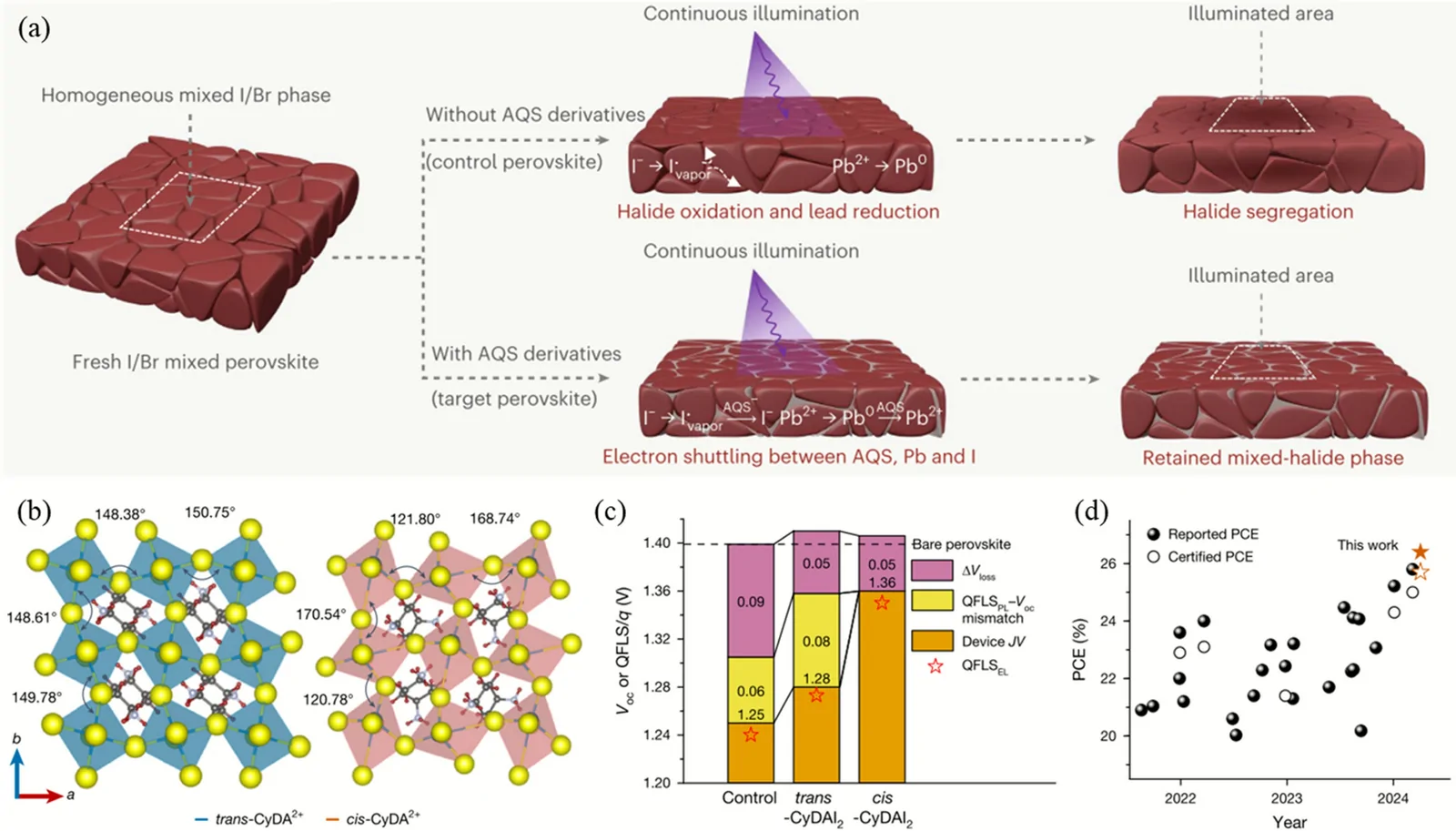
**a** Mechanism of the sustainable elimination of metallic Pb_0_ and I_0_ species in perovskite enabled by the redox mediator as additive, and its effect on suppressing halide segregation. Reproduced with permission from Ref. [128]. Copyright 2024, Springer Nature. **b** Calculated in-plane lattices structures of the trans-CyDAI_2_ and cis-CyDAI_2_-incorporated perovskite. **c** Summary of the voltage losses by comparing the quasi-Fermi-level splitting calculated from photoluminescence quantum yields. **d** Summary of the photovoltaic performance of the perovskite–organic TSCs reported in the literature. Reproduced with permission from Ref. [116]. Copyright 2024, Springer Nature

The migration of halogen ions and phase separation in WBG perovskites under illumination cause substantial energy losses, which constrain the performance of perovskite/organic tandem solar cells [135]. To mitigate this issue, Jen et al. developed a series of multifunctional redox mediators based on anthraquinone [128]. These mediators selectively reduce iodine and oxidize metallic lead while simultaneously passivating defects through tailored cation substitution. The synergistic effects of these mechanisms enable the fabrication of uniform and phase-stable WBG perovskite films under illumination (Fig. 11 a). For PSCs with a bandgap of 1.81 eV, a PCE of 19.58% was achieved, accompanied by a high *V*_OC_ of 1.35 V. The perovskite/organic tandem solar cells based on this approach demonstrated an PCE of 25.22% (certified at 24.27%) and exhibited operational stability with *T*_90_ > 500 h. Li et al. discovered that pseudo-halogen thiocyanate (SCN) ions can enter the perovskite lattice, forming an I/Br/SCN alloy and occupying iodine vacancies [127].

This strategy suppresses the migration and phase separation of halogen ions within the lattice and film. As a result, the perovskite/organic tandem solar cell achieved a high certified PCE of 25.06%. Moreover, due to interface recombination at the perovskite/C_60_ interface, WBG PSCs typically exhibit higher *V*_OC_ losses compared to conventional PSCs. To solve this problem, Li's group developed a novel surface passivation agent, cyclohexane 1,4-diammonium diiodide (CyDAI_2_), which naturally encompasses two isomer structures with ammonium groups located on either the same or opposite sides of the cyclohexane ring (denoted as *cis*-CyDAI_2_ and *trans*-CyDAI_2_, respectively) [116]. Calculations reveal that the in-plane Pb–I–Pb angles undergo notable variations in the perovskite film treated with *cis*-CyDAI_2_, whereas the corresponding angles in the film treated with *trans*-CyDAI_2_ vary within a more restricted range (Fig. 11 b). Further research revealed that passivation with *cis*-CyDAI_2_ reduces the mismatch between the quasi-Fermi levels (QFLs) and the *V*_OC_ in WBG PSCs with a bandgap of 1.88 eV, elevating their *V*_OC_ to 1.36 V (Fig. 11 c). The constructed monolithic perovskite/organic tandem solar cell exhibits a PCE of 26.4% (certified at 25.7%), which represents one of the highest PCEs reported for this type of device (Fig. 11 d).

## Commercialization Prospects

While laboratory-scale PSCs have demonstrated remarkable PCEs, rivaling those of traditional silicon-based counterparts, their transition to commercial viability hinges significantly on long-term operational stability, especially when scaling up to larger areas [12, 31, 136, 137]. Any degradation in stability can lead to reduced energy output, increased maintenance costs, and ultimately, a shorter lifespan, all of which are detrimental to the economic feasibility and market acceptance of perovskite technology. In the past two years, researchers delved deeply into the degradation mechanisms of PSCs, with a particular emphasis on intrinsic factors such as defects and ion migration. By employing targeted regulation strategies, high-performance PSCs demonstrated their capability to withstand rigorous testing conditions, including the damp-heat test, as well as standardized tests like ISOS-L-1I and ISOS-D-2I. Concurrently, researchers begun to turn their attention to the challenges faced by PSCs when operated under real-world outdoor conditions, such as perovskite decomposition induced by ultraviolet radiation and lattice strain caused by day–night cycles. For large-scale fabrication, the attainment of uniform, dense, and low-defect perovskite films is crucial. This necessitates innovations in deposition techniques, advancements in interface engineering, and breakthroughs in materials science. Furthermore, breakthroughs in technologies such as the utilization of green solvents, fabrication in ambient air, and kilogram-scale synthesis of perovskite precursors in aqueous phases are accelerating the commercialization of PSCs. PSCs with outstanding stability are summarized in Table 3.

**Table 3:** Summary of PSCs with outstanding stability performance

Device type	Area(cm^2^)	Initial PCE (%)	Test standard	Environment	Time (h)	Retention PCE (%)	References
Single, rigid	0.0412	26.14	MPPT	room temperature, ambient air	2500	97.2	[26]
Single, rigid	0.057	26.88	MPPT	ambient air, 65 °C	2000	98.2	[58]
Single, rigid	0.08	24.8	damp-heat	85 °C, 85 RH	1000	98.9	[75]
Single, rigid	0.057	26.21	MPPT	ambient air, 65 °C	2400	96.1	[61]
Single, rigid		25.1	MPPT	ambient air, 65 °C	2000	95	[92]
Single, rigid	0.0624	26.3	day/night cycles	room temperature to about 85 °C	576	96	[62]
Single, rigid	0.0625	24.6	MPPT	90 °C	3670	97.3	[138]
Single, rigid	0.16	23.3	MPPT	N_2_, room temperature	4500	99	[36]
Single, rigid	0.076	24.6	Repeated 12 h light on/off cycles	ambient air, 65 °C	10,000	98.35	[139]
Single, rigid	0.1	26.1	day/night cycles	–	1200	95	[42]
Single, rigid	1	25	MPPT	85 °C, 60 RH	2000	95	[55]
Single, rigid	11.7	22.69	MPPT	N_2_, 45 °C	3500	93	[140]
Single, rigid	17.88	18	MPPT	Outdoor	4872	88.9	[43]
Single, rigid	27.2	23.2	MPPT	85 °C, 85 RH	1900	87	[41]
Single, rigid	36	18.41	MPPT	85 °C, 50 RH	1000	95	[141]
Single, rigid	228	18.1	MPPT	ambient air, 40 °C, 46 RH	3000	~ 100	[48]
Single, rigid	715.1	22.80	ISOS-D-2	ambient humidity, 65 °C	528	94	[32]
Single, rigid	7906	15.0	Outdoor operation	Outdoor	1 year	98	[44]
Single, flexible	0.08	24.51	10,000 bending cycles	40 °C	–	91	[70]
Single, flexible	9	19	5,000 bending cycles	–	–	84	[71]
Single, flexible	900	16.4	1,000 bending cycles	–	–	96.2	[72]
Perovskite/silicon	1	31.46	damp-heat	85 °C, 85 RH	1000	90.2	[118]
Perovskite/silicon	25	29.4	MPPT	25°	1301	98.3	[121]
Perovskite/perovskite	1.05	27.5	MPPT	ambient air, 45 °C	1000	90	[125]
Perovskite/perovskite	20.25	24.5	MPPT	ambient air, 50 °C	656	80	[47]
Perovskite/organic	0.0058	25.82	MPPT	N_2_, 45–55 °C	1000	85	[127]
Perovskite/organic	0.09	26.4	MPPT	N_2_	700	93	[116]

When the device area is scaled up, defects become more non-uniformly distributed and the perovskite film exhibits increased inhomogeneity. A commonly observed phenomenon is the concurrent deterioration of both the PCE and stability. The non-uniform degradation at scale is a complex issue with multiple root causes [3, 142]. One major factor is the inherent heterogeneity in large-area perovskite films. During the fabrication of large-area devices, slight variations in deposition conditions, such as temperature gradients, solvent evaporation rates, and precursor distribution, can lead to non-uniform film quality. These local variations in the perovskite layer can initiate different degradation pathways. Another significant root cause is related to the interface properties. In large-scale devices, the interfaces between the perovskite layer and the adjacent CTLs may not be as uniform as in small-area devices. Non-ideal interface contact can result in charge accumulation at certain regions, which can accelerate degradation processes. Moreover, the mechanical stress distribution in large-area devices is different from that in small-scale ones. As the device area increases, the thermal and mechanical stresses generated during operation or under environmental changes (such as temperature fluctuations) are more likely to be non-uniformly distributed. This non-uniform stress can cause micro-cracks or delamination in the perovskite film or at the interfaces, which in turn can act as pathways for moisture and oxygen penetration, exacerbating the degradation.

### Stability

**Fig. 12:**
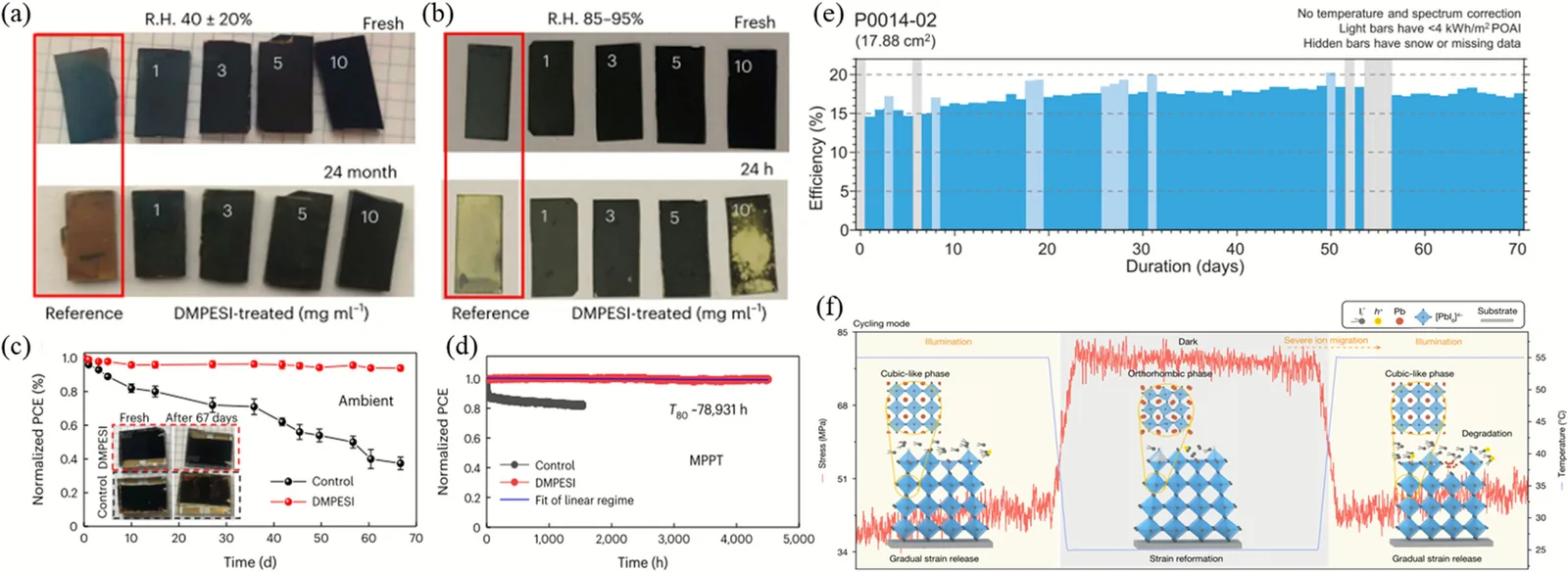
**a** Photographs of fresh and 24-month-aged unencapsulated perovskite film without and with DMPESI treatment of different concentrations (from left to right: reference, 1 mg mL^− 1^, 3 mg mL^− 1^, 5 mg mL^− 1^, 10 mg mL^− 1^) in ambient air with RH. 40 ± 20%. **b** Photographs of fresh and 24 h aged unencapsulated perovskite film without and with DMPESI treatment of different concentrations (from left to right: reference, 1 mg mL^− 1^, 3 mg mL^− 1^, 5 mg mL^− 1^, 10 mg mL^− 1^) in high humid condition of RH. 85%∼95%. **c** Dark shelf stability of unencapsulated control and DMPESI-treated PSCs and inserted photos are the devices before and after aging in ambient condition with RH around 20–40%. **d** Long-term operational stability of the unencapsulated control and treated devices under MPPT with continuous 1-sun illumination under N_2_ flow at room temperature. Reproduced with permission from Ref. [36]. Copyright 2024, Springer Nature. **e** Outdoor test of hybrid HTM–based perovskite minimodule measured by PACT center. Reproduced with permission from Ref. [43]. Copyright 2024, AAAS. **f** Illustration of the degradation mechanism of perovskite crystal in the cycling mode. Perovskite film stress was calculated from curvature measurements. Reproduced with permission from Ref. [62]. Copyright 2024, Springer Nature

Ion migration within perovskite films represents a pivotal factor influencing the operational stability of PSCs, yet a comprehensive understanding of the associated degradation mechanisms has been lacking [143]. Stolterfoht et al. reveal that mobile ion-induced internal field screening is the dominant factor in the degradation of PSCs under operational conditions [144]. Furthermore, they identify the mobile ion density as primarily responsible for *V*_OC_ loss and device aging. Hagfeldt et al. introduce a sulfonium-based molecule, dimethylphenethylsulfonium iodide (DMPESI), for post-deposition treatment of FAPbI_3_ films [36]. By precisely controlling the dosage of DMPESI, the unencapsulated FAPbI_3_ films can retain their pure black phase for a duration of two years when exposed to ambient air (Fig. 12 a). Even in high humidity conditions with RH of 85–95%, the optimized samples maintain extended stability, continuing to perform well after the control sample has undergone complete degradation (Fig. 12 b). Therefore, the PSCs treated with DMPESI demonstrated excellent stability in various aging tests. After being stored in ambient air for 67 days, the PCE of the control PSC decreased by ~ 60%, whereas the treated PSC maintained 94% of its initial PCE value (Fig. 12 c). Encouragingly, after undergoing 4500 h of MPPT, the treated PSC exhibited only a minimal decrease in PCE of less than 1% (Fig. 12 d). This represents one of the most stable and efficient PSCs reported to date. Feng and colleagues report a novel living passivation strategy that employs a hindered urea/thiocarbamate bond Lewis acid–base material (HUBLA) [35]. When exposed to moist or thermal environments, HUBLA triggers the generation of novel agents, which facilitate the dynamic repair of defects within perovskite films. This passivation strategy led to the realization of high-performance devices with a PCE of 25.1%, which retains 88% of its initial PCE after aging for 1000 h at 85 °C and 30% RH in air. The lattice deformation and structural evolution of perovskite thin films under the combined effects of electric field, temperature, and light illumination severely limit the operational durability of PSCs. In light of this challenge, Yang et al. improved the mechanical properties of perovskite thin films by incorporating a polymer-coupled monolayer graphene interface [138]. The synergistic interaction between graphene and the polymer mitigated the phenomenon of light-induced lattice expansion. As a result, the deformation ratio was dramatically reduced from 0.31–0.08%. Meanwhile, the modulus and hardness of the perovskite thin films were doubled. When exposed to an ambient temperature of 90 °C and undergoing MPPT for over 3670 h, the device still retained more than 97% of its initial PCE. Li et al. promoted the controlled growth of 1D and 2D structures on 3D perovskites via dimensional engineering [141]. The differences in the electrostatic potential distribution and the spatial effects of intermolecular forces among heterogeneous ligands lead to the formation of distinct low-dimensional perovskites. Employing this strategy, the PSM (6 × 6 cm^2^) maintained around 95% of its initial PCE after 1000 h of ISOS-L-3 accelerated aging tests, which was one of the highest stabilities reported for n-i-p structured modules.

The heterointerfaces between perovskite films and CTLs are prone to mechanical failure and chemical degradation. These issues have a marked impact on the long-term stability of PSCs, particularly under conditions of thermal cycling and damp heat [145]. Zhou et al. introduced *R*-/*S*-methylbenzyl-ammonium between the perovskite absorber layer and the ETL, thereby constructing an elastic yet strongly bonded chirality-mediated heterointerface [146]. This interface leverages enantiomer-controlled entropy to enhance tolerance against fatigue and material degradation induced by thermal cycling, thereby improving mechanical reliability. Furthermore, the heterochiral arrangement of organic cations results in closer packing of benzene rings, which enhances chemical stability and charge transfer. The encapsulated PSCs exhibited a retention of 92% of their PCE under a thermal cycling test (from − 40 to 85 °C; 200 cycles over 1200 h) and 92% under a damp-heat test (85% RH, 85 °C, 600 h). Hou et al. conducted a comprehensive analysis of the behavior of heterointerfaces in PSCs during crystal growth and aging stages through an effective debonding technique [40]. The results revealed a strong correlation between interface bonding, proton transfer interactions, and degradation. That is, there exists a trade-off between mechanical and chemical stability of the devices. To address this, they incorporated a blend of Me-4PACz and 9*H*,9'*H*-[3,3'-bicarbazole]9,9'-diylbis(butane-4,1-diyl))diphosphonic acid (DCZ-4P) molecules, which introduced additional phosphonic acid anchoring groups. This strategy enhanced the adhesion at the interface between the metal oxide and perovskite. The devices achieved a high PCE of 25.6% and maintained 90% of their initial performance after 1000 h of testing under the ISOS-L-1I and ISOS-D-2I standard test protocols. Moreover, they retained 95% PCE after 500 cycles, which surpasses the standards set by IEC 61215 and ISOS-T-3I.

While some PSCs have achieved *T*_90_ lifetimes exceeding 10,000 h under illumination, it is crucial to note that most of these tests utilize inorganic light-emitting diodes (LEDs) as light sources [147, 148]. In comparison with LEDs, natural sunlight contains a higher concentration of UV light. Consequently, the operational stability of perovskite devices tends to be compromised when deployed outdoors compared to indoor environments. Huang et al. observed that under UV illumination, weak chemical bonding between the perovskite/HTM/TCOs results in accelerated migration of A-site cations [43]. To address this, they introduced an aromatic phosphonic acid, [2-(9-ethyl-9H-carbazol-3-yl)ethyl]phosphonic acid (EtCz3EPA), at the bottom interface of the device, serving as an interface binder between the perovskite and the substrate. This strategy enhanced interface bonding and suppressed UV-induced degradation. Based on this approach, a PSC minimodule maintained an operational PCE of over 16% after 29 weeks of outdoor testing conducted at the Perovskite PV Accelerator for Commercializing Technologies (PACT) center (Fig. 12 e). Yuan et al. discovered that I_2_ typically diffuse considerable distances before causing damage to the perovskite under illumination [149]. Fluorodecyl iodide, leveraging directed I_x_^−^ affinity through halogen bonding, effectively traps and confines I_x_ within the perovskite layer. Inverted PSCs based on perfluorodecyl iodide exhibited more than tenfold enhanced UV radiation and thermal light stability at 85 °C and 1 sun illumination. Additionally, they demonstrated over 1000-fold improved reverse bias stability under ISOS-V aging tests. Due to lattice strain resulting from thermal expansion and contraction, efficient FAPbI_3_ PSCs degrade significantly faster under natural day–night cycling conditions (Fig. 12 f). Li et al. introduced phenylselenium chloride (Ph-Se-Cl) to regulate lattice strain in the perovskite during day–night cycling [62]. After modification, the devices achieved a certified PCE of 26.3%, and their *T*_80_ lifetime under cycling conditions was prolonged by 10 times. Qin et al. observed that, under dark/light alternating conditions, the migration of lithium within the HTL can rapidly cause the degradation of α-phase perovskite [42]. To tackle the instability issues associated with such light–dark cycling, they replaced the lithium dopant with a methylammonium dopant. By employing this strategy, the device retained 95% of its initial PCE after undergoing 1200 h of continuous light–dark cycling and 3000 voltage on/off cycles.

### Scalable Fabrication

**Fig. 13:**
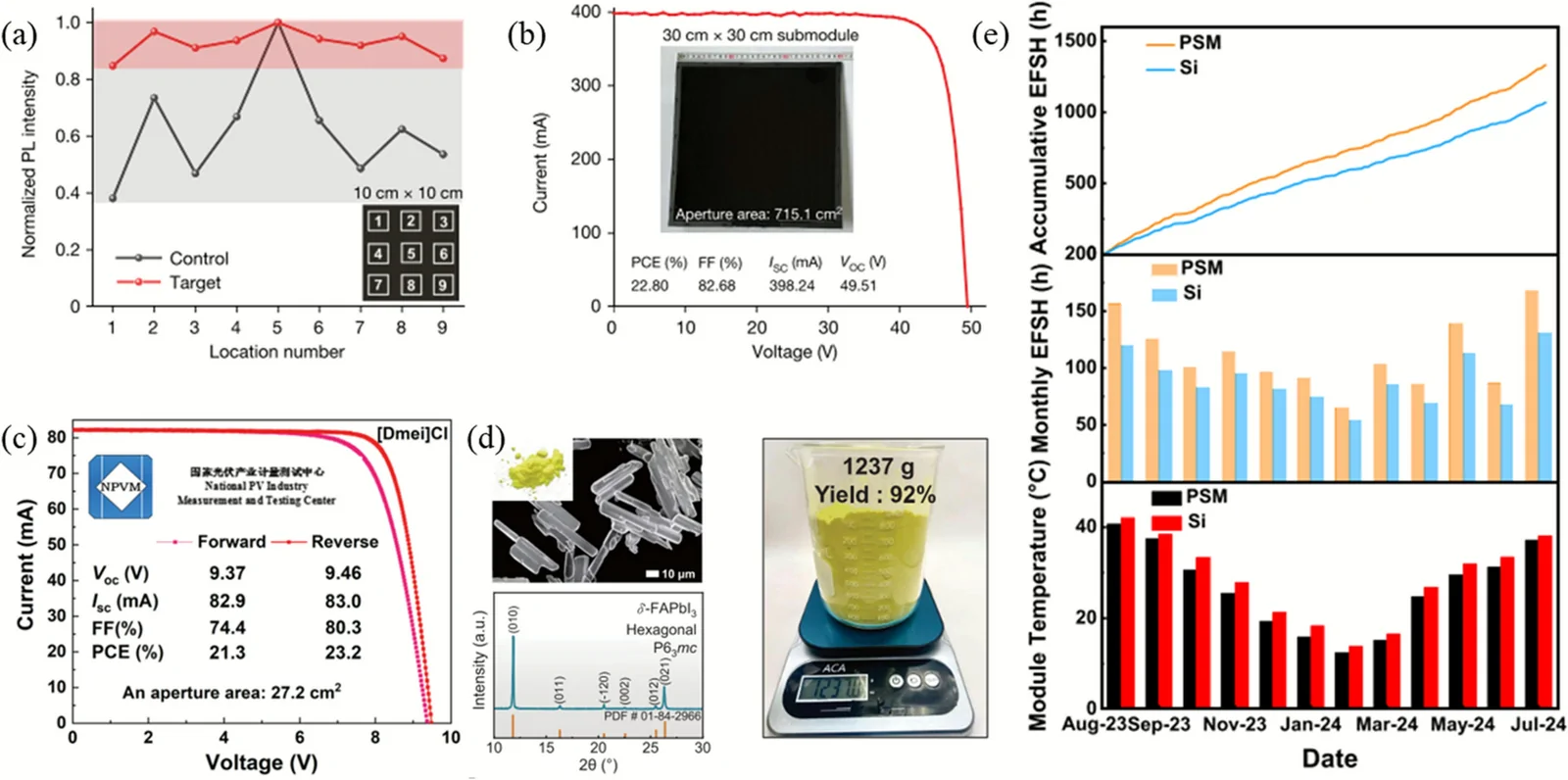
**a** Normalized photoluminescence intensity of 10 × 10 cm^2^ perovskite films when measured at different positions. **b** *J*–*V* curve of the champion 30 × 30 cm^2^ target PSM and photograph (inset) of a 30 × 30 cm^2^ PSM. Reproduced with permission from Ref. [32]. Copyright 2024, Springer Nature. **c** Certified *I*-*V* curves from forward and reverse scans of a PSM with an aperture area of 27.2 cm^2^. Reproduced with permission from Ref. [41]. Copyright 2024, AAAS. **d** Scanning electron microscopy images (top), X-ray diffraction pattern (bottom) and photograph (Right) of large-scale synthesized FAPbI_3_ microcrystals. Reproduced with permission from Ref. [51]. Copyright 2024, AAAS. **e** Comparison of energy output between PSM and silicon-based modules during outdoor operation. Reproduced with permission from Ref. [44]. Copyright 2025, AAAS

The uneven distribution of defect sites is a pivotal factor contributing to the decline in PCE when scaling up the area of perovskite photovoltaic devices [150]. Zhao et al. introduced a functional cation, 2-(1-cyclohexenyl)ethyl ammonium, into FAPbI_3_ to construct high-mobility 2D perovskites that horizontally cover the surface and vertically penetrate the grain boundaries of 3D perovskites [32]. This not only converts PbI_2_ and δ-FAPbI_3_ impurities into stable 2D perovskites, achieving uniform defect passivation, but also provides interconnected channels for efficient carrier transport. Consequently, a notable enhancement in the uniformity of interface contact across various regions of large-area perovskite thin films was achieved (Fig. 13 a). Based on this impurity-healing interface engineering strategy, the fabricated PSM achieved a certified PCE of 22.46% with a high FF of 81.21% on an aperture area of 715.1 cm^2^ (Fig. 13 b). Bu et al. observed chain length dependence and halide-related phase segregation issues when 2D perovskites grow on the surface of 3D perovskites [49]. By treating the perovskite layer with formamidinium bromide containing long-chain (> 10) alkylamine ligand salts, a large-area, uniform 2D perovskite passivation layer can be obtained. Guo et al. performed fluorine passivation on the perovskite active layer through vapor deposition [48]. This method enabled PSMs (228 cm^2^) to achieve a PCE of 18.1%, with an estimated *T*_80_ lifetime of 43,000 ± 9000 h under accelerated aging at 30 °C under 1-sun illumination, which is comparable to the stability of the best-performing small-area devices.

Nazeeruddin et al. report on a synergistic strategy that combines dopant and additive [69]. Specifically, they utilize MACl as the dopant alongside a Lewis-basic ionic-liquid additive, namely 1,3-bis(cyanomethyl) imidazolium chloride ([Bcmim]Cl). This approach successfully mitigates the degradation of perovskite precursor solutions and suppresses the aggregation of MACl. Consequently, it facilitates the formation of phase-uniform, stable perovskite films with high crystallinity and reduced defects. Through this synergistic regulation strategy, PSMs achieved a certified PCE of 22.97% on an aperture area of 27.22 cm^2^. The same research group, by incorporating *N*,*N*-dimethylmethyleneiminium chloride into the perovskite precursor solution, formed dimethylammonium cations and the previously unobserved methyl tetrahydrotriazinium ([MTTZ]^+^) cations, effectively enhancing the perovskite films [41]. The optimized PSMs achieved a certified PCE exceeding 23.2%, with a stable PCE maintained at 23.0% (Fig. 13 c). This represents a new record for the highest certified PCE among PSMs at the time of this report. Xu's team conducted in-depth research on the purification mechanism of perovskite crystals synthesized in aqueous phases and successfully obtained high-purity FAPbI_3_ crystals with an average purity of 99.994% through this method [51]. This achievement enabled kilogram-scale production using low-cost, low-purity raw materials, with costs two orders of magnitude lower than those of commercial PbI_2_ and methylammonium iodide (Fig. 13 d). This study not only established solvent screening criteria for perovskite crystal synthesis but also developed an evaluation method for perovskite crystal raw materials.

Recently, some novel techniques for preparing perovskite thin films have begun to show their potential in the large-scale fabrication of devices. Tan et al. proposed a marker-pen-based fabrication technique for perovskite thin films [151]. By precisely regulating the ink concentration, writing pressure, moving speed, and tip width, and leveraging the fibrous capillary structure of the marker pen, they achieved effective control over the homogenization of perovskite colloid size, thin film thickness (ranging from 200 nm to over 1000 nm), and pattern area (from 1 cm^2^ to over 100 cm^2^). Furthermore, through solvent engineering design, they were able to directly write crystalline perovskite thin films at room temperature. By employing the marker-pen writing technique, PSMs featuring carbon electrodes have achieved PCEs of 16.3% and 14.5% on rigid and flexible substrates, respectively. It is worth mentioning that this approach does not require photomasks or laser processing. To address the challenge that the nucleation and growth of perovskites are highly sensitive to processing methods, Jen et al. developed a temperature-controlled vacuum quenching (T-VAQ) method combined with in situ photoluminescence spectroscopy monitoring [140]. This method regulates the nucleation process by reducing the vacuum quenching temperature, thereby broadening the time window for post-treatment and successfully fabricating high-quality large-area perovskite films. Ultimately, PSMs based on this method achieved a PCE of 22.69% (certified 21.60%) over an aperture area of 11.7 cm^2^. Moreover, the corresponding devices maintained over 93% of their initial PCE after continuous operation for 3500 h under 1 sun illumination at 45 °C. Yao et al. developed a laminar airflow dryer (LAD) to assist in the crystallization of perovskite thin films [44]. The LAD integrates the precision of spin-coating and the uniformity of vacuum flash evaporation, enabling rapid and uniform drying of large-area perovskite thin films through 3D laminar flow. Based on the LAD technology, the certified PCE of the PSM with an area of 0.7906 m^2^ has achieved 15%. After one year of outdoor testing, their energy yield was 29% higher than that of silicon-based modules (Fig. 13 e), with a first-year degradation rate of less than 2%, and a projected lifespan of up to 9 years.

## Conclusion and Outlook

In summary, PSCs have witnessed remarkable advancements over the past two years. The PCEs of single-junction devices and two-terminal tandem devices have surpassed 27% and 34%, respectively. This progress is accompanied by substantial improvements in device stability and significant strides in large-scale manufacturing. These collective advances have narrowed the gap between laboratory prototypes and industrialization, positioning PSCs at the threshold of commercialization. Ongoing research focuses on further enhancing the PCE of PSMs and prolonging their stability for outdoor operations. Furthermore, greater attention should be given to the environmental-friendliness of perovskite materials and related solvents, as well as the recycling and reuse of PSCs. The following suggestions are proposed for the future development of PSCs:

1. Currently, PSCs have achieved relatively high levels in terms of their *V*_OC_ and FF. However, their *J*_SC_ lingers around ~26 mA cm^− 2^, still lagging behind the Shockley–Queisser limit for perovskite semiconductors, which stands at 27.3 mA cm^− 2^ for an *E*_g_ of 1.55 eV and 28.69 mA cm^− 2^ for an *E*_g_ of 1.5 eV [152]. For comparison, the best crystalline silicon solar cells achieved a *J*_SC_ of 42.35 mA cm^− 2^, representing 95.8% of its theoretical Shockley–Queisser limit of 44.2 mA cm^− 2^ [8]. Therefore, there is considerable room for advancing the PCE of PSCs by focusing on the enhancement of the *J*_SC_. In light of their inherent structural and material characteristics, PSCs suffer from the following optical losses: (a) parasitic absorption losses arising from TCO, HTL, as well as various passivation layers and dopants; (b) reflection losses at surfaces and interfaces due to differences in refractive indices among the multiple thin-film materials stacked within the PSCs; (c) insufficient absorption of photons in the long-wavelength range, particularly those within the 750–850 nm band, due to inadequate thickness of the active layer. Given these factors, the viable future directions for PSCs research lie in the development of transport materials that exhibit both low absorption and excellent conductivity, as well as enhancing light management through micro- and nano-photonic structures.
2. Although small-area PSCs demonstrated impressive PCEs and operational stability, both of these attributes undergo notable decline when the device area is scaled up. These discrepancies pose a formidable obstacle on the journey toward the commercialization of PSCs. Notably, such a discrepancy is not pronounced in crystalline silicon solar cells. Despite the inherent defect tolerance of perovskite materials, defects are still primarily responsible for device performance degradation. When subjected to external stimuli such as water, oxygen, light, heat, and mechanical stress, these defects serve as the primary sources of device degradation. For small-area cells, current thin-film crystallization modulation and passivation strategies can effectively mitigate the adverse impacts of defects. However, as the cell area increases, the formation and distribution of defects become uncontrollable. Therefore, future research should focus on developing scalable deposition techniques that maintain high film quality and uniformity. Furthermore, the development of passivation layers that are compatible with device area, dense, and uniform should also garner attention from researchers in the community of PSCs. In addition to internal defect regulation, encapsulation can physically isolate the device from the erosion of the external environment, thereby enhancing its operational stability [153, 154]. However, from an optical perspective, the encapsulation layer introduces additional absorption and reflection losses of light, which consequently sacrifices part of the device's PCE. Therefore, the development of high-performance encapsulation materials holds great promise for promoting the commercialization of PSCs [155, 156].
3. On the eve of commercialization, the recycling of materials utilized in PSCs has emerged as critical areas of focus. Recycling minimizes material waste and offers a sustainable solution to the disposal of end-of-life PSCs, thereby positively contributing to environmental conservation efforts. While the recycling processes for traditional silicon-based solar cells are well established, those for PSCs are still in their nascent stages. From an economic perspective, the recycling of TCO and metal electrodes offers the highest return on investment. However, to ensure that devices based on recycled materials achieve a PCE comparable to that of devices based on fresh materials, it is crucial to enhance the recycling purity of materials such as perovskites and CTLs [157]. Recent research has focused on developing aqueous-based recycling methods for PSCs. This approach enables the recycling of all components in PSCs without relying on environmentally hazardous solvents. The recycling efficiency reaches 99.0%, and it leads to a 96.6% reduction in resource depletion and a 68.8% decrease in human toxicity (cancer effects) impacts [158]. Another approach involves layered recycling techniques, which separate and recover different components of the PSC through a series of steps. This method often includes thermal treatment to soften encapsulating materials, followed by layered recovery of individual components. Based on this strategy, the estimated recyclable mass fraction reaches as high as 99.97%. In laboratory settings, recycling can cut material costs by up to 63.7%, while in industrial manufacturing, the cost reduction can be as much as 61.4% [159]. Compared to landfill disposal, these recycling strategies significantly reduce lead pollution, alleviate resource depletion issues, and lower the levelized cost of electricity (LCOE) for PSCs [160]. Additionally, efforts are underway to develop lead-free perovskite alternatives, which could potentially simplify recycling processes and enhance environmental compatibility.
4. The integration of AI, particularly ML, has shown tremendous promise in advancing PSC research. By leveraging ML algorithms, AI can rapidly process and analyze massive experimental datasets, enabling the identification of optimal material combinations and processing conditions that maximize PCE and device stability. AI-driven predictive models can forecast the performance of PSCs under diverse environmental factors, guiding researchers in refining device architectures. Additionally, AI-powered image analysis techniques can precisely detect and classify defects in perovskite films, such as pinholes or grain boundaries, which are critical for improving film quality and reducing recombination losses. Furthermore, AI can optimize the design of novel interface layers and additives by simulating their interactions with perovskite materials, accelerating the discovery of high-performance PSCs. As datasets grow larger and more comprehensive, AI models will become more accurate and reliable. Additionally, advancements in model optimization techniques, such as transfer learning and deep learning, will enable AI to achieve higher prediction accuracy with smaller datasets, addressing the challenge of limited data availability.

Finally, the LCOE of PSCs serves as a crucial indicator for determining their commercial viability. When the PCE and operational lifetime of PSC modules reach 20% and 7 years, respectively (31% and 8 years for perovskite-silicon tandems; 30% and 8 years for four-terminal perovskite-silicon tandems), they can attain an LCOE of 7.75 Cents (kW h)^− 1^, thereby making them commercially competitive with silicon technology [161]. Currently, the manufacturing cost and the LCOE for PSCs are estimated to be 0.57 $ W^− 1^ and 18–22 Cents (kW h)^− 1^, respectively. Among these costs, the materials cost accounts for 70% [160]. With breakthroughs in operational stability and material recycling, among other aspects, the future of PSCs looks promising, with the potential to revolutionize the way we harness and utilize solar energy.
